# Deep-learning-based sub-meter urban construction-site mapping reveals China’s dual-track urban renewal

**DOI:** 10.1093/nsr/nwag141

**Published:** 2026-03-10

**Authors:** Jiayi Li, Wenrui Wang, Xin Huang, Xiaofeng Pan, Song Ma, Bin Chen, Liangpei Zhang

**Affiliations:** School of Remote Sensing and Information Engineering, Wuhan University, Wuhan 430079, China; State Key Laboratory of Information Engineering in Surveying, Mapping and Remote Sensing, Wuhan University, Wuhan 430079, China; School of Remote Sensing and Information Engineering, Wuhan University, Wuhan 430079, China; Shenzhen Ecological and Environmental Monitoring Center of Guangdong Province, Shenzhen 518049, China; Shenzhen Ecological and Environmental Monitoring Center of Guangdong Province, Shenzhen 518049, China; Future Urbanity & Sustainable Environment (FUSE) Lab, Division of Landscape Architecture, Faculty of Architecture, The University of Hong Kong, Hong Kong 999077, China; State Key Laboratory of Information Engineering in Surveying, Mapping and Remote Sensing, Wuhan University, Wuhan 430079, China

**Keywords:** China’s new-type urbanization, urban construction sites, sub-meter mapping, deep-learning framework

## Abstract

China’s New-Type Urbanization Plan since 2015—the world’s largest urbanization endeavor—reshapes the nation’s socioeconomic landscape but lacks high-precision, fine-scale progress monitoring. Urban construction sites (UCSs)—barometers of urban spatial expansion and renewal—offer a detailed observational window. A sub-meter-resolution deep-learning framework for nationwide UCS mapping is proposed. Using a Segment Anything Model-enabled weakly supervised method for pixel-level UCS annotation, a spectral–texture dual-branch segmentation network with 94.3% overall accuracy identifies 541 177 UCSs (including 10-m² micro-sites) across 372 cities. K-means clustering partitions cities into four typologies, uncovering a dual-track parallel pattern (incremental expansion + stock optimization) vs the classical ‘growth–decline–renewal’ trajectory. Spatial analysis shows that UCS construction correlates with annual PM₂.₅ concentrations; green dust-proof net coverage (<10%) fails to curb pollution. The framework serves as a ‘microscope’ for evaluating new-type urbanization and supports sustainable planning.

## Teaser

High-resolution GeoAI tracks China’s urban construction down to 10 m2, uncovering a dual-track transition from incremental expansion to strategic stock optimization through sub-meter satellite data.

## INTRODUCTION

In recent decades, the extraordinary speed and scale of China’s urbanization have been witnessed, with rapid land expansion and extensive construction projects [1,2]. Under the principle of ‘people-centered and sustainable urbanization’, the government launched the National New-type Urbanization Plan in 2014, designating pilot cities for the initiative [3,4]. Subsequently, China officially entered a new phase of comprehensive urban renewal, calling for a shift ‘from incremental expansion to stock-oriented renewal’ and favoring small-scale, phased, quality-centered redevelopment [5,6]. Yet cities differ markedly in development stage, demography, fiscal capacity and spatial form [7,8]; implementation therefore involves a complex mix of goals, capabilities and constraints, precluding a single template. The success—or failure—of urban renewal will resonate across several UN Sustainable Development Goals (SDGs), notably sustainable cities (SDG 11), good health and well-being (SDG 3) and climate action (SDG 13) [9]. However, population-based monitoring—the mainstream approach in urban-renewal research [10,11]—faces two key limitations: (i) the Hukou system’s registration–residence disparity creates significant gaps between official and actual populations; (ii) demographic statistics lack the resolution and frequency to track high-frequency renewal dynamics, necessitating fine-scale monitoring frameworks. This underscores the need for high-precision, fine-grained and reliable monitoring approaches for China’s New-Type Urbanization. In contrast, urban construction sites (UCSs) offer a sensitive gauge of urban transformation: each logs the precise location, timing and scale of land clearance, infrastructure installation and new building, alongside their environmental impacts, revealing capital flows and spatial reconfiguration [12–14]. In renewal-dominated contexts, swarms of fragmented micro construction activity create short-cycle, high-frequency disturbances that often evade existing monitoring systems, forming governance blind spots [15]. A nationwide, sub-meter atlas of construction activity would allow the systematic comparison of divergent renewal trajectories, identify hidden environmental and social risks, and underpin adaptive, city-specific management strategies.

During the 14th Five-Year Plan period (2021–2025), China proposed to build a ‘smarter, greener, safer’ construction industry [16] and mandated the use of green dust-proof nets (DPNs) [17–19]. The primary task of UCS monitoring is to regularly identify each UCS parcel, confirm its location and assess its exposed‐area ratio, which requires enormous labor-intensive manual surveys [12]. To date, there has been a lack of nationwide UCS records. Regarding the temporal dynamics of UCSs, it is also imperative to implement a timely UCS-monitoring approach. In recent decades, the satellite remote-sensing technique has made some progress in Earth observation [15,20–22]. Several works have been developed to monitor UCSs from three main perspectives [12,23,24]: spatial resolution and coverage, algorithm development and sample collection. Notably, in today’s context of urban renewal, many UCSs are small in area and scattered throughout cities [25]. Large-scale mapping efforts often rely on medium- to low-resolution remote-sensing imagery (e.g. 10 m), which tends to overlook these diminutive sites [12,26,27]. In contrast, while high-resolution unmanned aerial vehicle (UAV) imagery can capture fine details [13,28–30], its application is constrained by high acquisition costs, rendering it impractical for extensive monitoring [31]. Moreover, due to the various stages of construction and the inherent randomness of site operations, UCSs exhibit significant instability and complexity (see Fig. S4c–e). Shallow machine-learning methods (e.g. random forest), limited by their simplistic model structures, struggle to capture the intricate patterns within UCS samples, resulting in inferior performance [32]. Although deep-learning-based approaches offer enhanced capabilities, their performance is heavily dependent on the scale and diversity of the available annotated UCS datasets [33]. Currently, the number of publicly available UCS samples is very limited [17,34]. Thus, the absence of a comprehensive large-scale UCS dataset poses a significant challenge for researchers and institutions aiming to advance monitoring and regulatory efforts.

To fill this gap, we develop a deep-learning framework to detect nationwide UCSs in a labor-efficient and replicable manner. Under the era of deep learning, artificial intelligence for geoscience has achieved several remarkable successes, with the aid of big data [35,36]. Benefitting from this, we carry out UCS-monitoring work from both the data and model perspectives. To balance image accessibility with spatial granularity, it is suggested that sub-meter satellite imagery from Google Earth could effectively support large-scale UCS monitoring. We categorized UCSs into three exposure-based types [12], namely DPN-covered (fully netted), exposed (un-netted) and mixed (partially netted), at various scales by using an efficient semi-automated deep-learning process. This process only requires several initial image-level annotations (i.e. indicating whether the image contains UCSs) to label UCSs with pixel-level locations. The dataset contains 217 810 UCSs covering approximately 43 000 km^2^ of satellite images across 372 Chinese prefecture-level and provincially administered cities (shortened to prefecture-level cities in this article) (Fig. S4a) with a resolution of 0.5 meters per pixel. Fig. S4f–h illustrates the appearance of three types of UCSs and Fig. S4b shows the proportion of the three types. Both the designed semi-automated labeling approach and the large-scale dataset can assist related researchers and government to conduct better UCS monitoring, supervision and regulation. Then, we model the texture and spectral characteristics of UCSs into a deep semantic segmentation network and propose a novel UCS-detection method. Experimental results confirm that the proposed model achieves the detection of 541 177 UCSs throughout China. More advantages of the proposed model over existing works can be found in Table S2. Using high-spatial-resolution satellite imagery (circa 2020), we developed China’s first open-source 0.5-meter-resolution UCS map. Spatial analysis of this nationwide inventory reveals that, during the current New-type Urbanization phase, the multiscale coordinated differentiation of urban construction spaces constitutes the foundational organizational framework of China’s urban-renewal logic. Concurrently, most cities, including 205 stock renewal transition cities and 59 integrated renewal cities, exhibit simultaneous peripheral expansion and core renewal, which diverge from the renewal patterns observed in Western countries. Notably, construction activities are significantly correlated with annual increases in PM_2.5_ concentrations. The current DPN coverage rates (≤10%) remain substantially below policy mandates, while nighttime construction operations remain poorly regulated, resulting in non-negligible lighting pollution.

## RESULTS

### UCS mapping from a spectral–texture dual-branch semantic segmentation model

This study introduces an efficient sample-collection method, generating annotations for 163 000 images nationwide by using 3000 DPN-covered images (with DPN-covered pixels), 3000 exposed images (with exposed area pixels) and 30 000 background images (without DPN-covered or exposed pixels). The pixel-wise annotations include DPN-covered, exposed, building, background and uncertain pixels. The visualization and analysis of the 163 000 nationwide images are provided in Fig. S4 and Method S1. Each image spans 1024 × 1024 pixels at 0.5-meter resolution, with UCS boundaries extracted via morphological post-processing (e.g. Fig. S4f–h; see the ‘Methods’ section for details). Considering the unique spatial and spectral characteristics of the DPN-covered and exposed areas, we developed a spectral–texture dual-branch semantic segmentation model (STTBNet) to classify DPN-covered, exposed, building and background pixels. The semi-automatically collected dataset comprising 163 000 images with pixel-level annotations was divided into training and validation sets at a 9:1 ratio. This model was applied across urban areas nationwide, followed by a post-processing step that utilized multi-source geographic information to aggregate the relevant pixels (DPN-covered/exposed pixels and their surrounding areas) into UCS parcels (details provided in the ‘Methods’ section). The nationwide UCS map achieved an overall accuracy (OA) of 94.3% (Table 1), indicating that the developed national-scale product is highly reliable and suitable for practical applications. The superiority of the STTBNet over current studies can be seen in Table S2 and Fig. S7.

**Table 1.** Performances of the STTBNet model and the nationwide UCS map.

STTBNet			Nationwide map		
**Exposed areas**	Precision	83.7%	**Exposed UCS**	Precision	82.7%
	Sensitivity	84.5%		Sensitivity	88.1%
**DPN-covered areas**	Precision	94.6%	**DPN-covered UCS**	Precision	91.8%
	Sensitivity	91.3%		Sensitivity	86.6%
**Building**	Precision	94.7%	**Mixed UCS**	Precision	89.9%
	Sensitivity	91.0%		Sensitivity	80.9%
**Background**	Precision	94.8%	**Background**	Precision	96.2%
	Sensitivity	95.4%		Sensitivity	97.1%
	OA	93.1%		OA	94.3%

**Figure 1.**
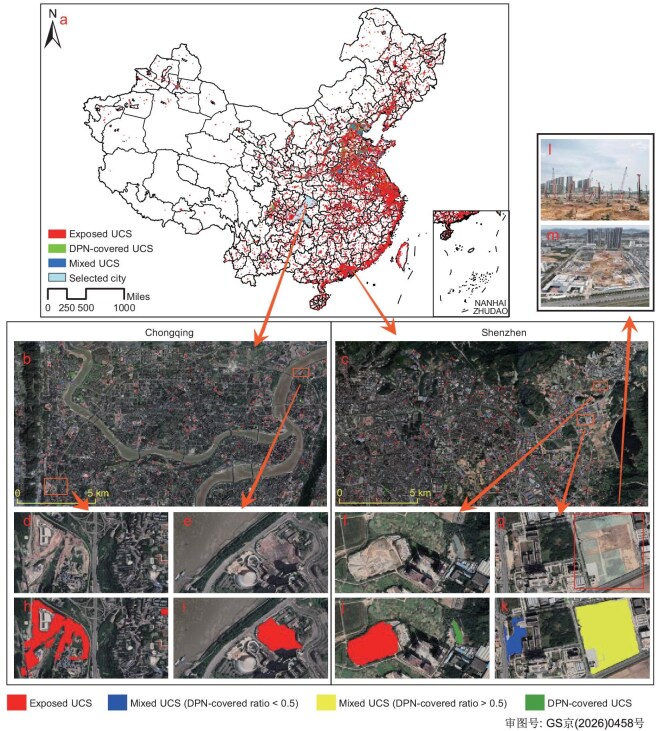
Visualization and validation of UCS-detection results. (a) Nationwide geographical distribution of UCS-detection results. (b, c) UCS distributions in selected regions of Chongqing and Shenzhen, highlighted by dots. (d–g) Photographs of UCSs in Chongqing’s urban core and fringe, and Shenzhen’s urban new town. (h–k) UCS masks from the nationwide UCS map, displaying exposed UCSs, DPN-covered UCSs, and mixed UCSs with varying DPN coverage ratios. (l, m) Street-view and bird's-eye-view images of a mixed UCS with high DPN coverage (highlighted in the box in g) in Shenzhen’s new town region. Image data © Google.

Figure 1 illustrates the reliability and accuracy of our detection results. The sub-meter-resolution data demonstrate high precision and detail retention in detecting UCSs in both Chongqing and Shenzhen areas (Fig. 1d–k). The error analysis (details provided in Method S2) as well as the Monte-Carlo dropout protocol on uncertainty quantification (details provided in Method S2) indicate balanced, high performance across the categories and overall reliability sufficient for nationwide-scale application. From the uncertainty figures (Fig. S11), the overall predictive uncertainty is low to moderate and spatially concentrated along class boundaries (exposed vs. DPN) and in micro/fragmented objects, with limited influence on city-level aggregates and the derived typology. The proposed work effectively distinguishes UCSs of different sizes and categories, validating its adaptability to diverse landscapes (see more illustrations in Figs S17–S26). On a national scale, exposed UCSs account for a significant proportion (Fig. 1a), whether in urban core areas (e.g. Fig. 1b) or in new towns and urban fringes (e.g. Fig. 1b and c), with only limited spatial coverage of DPN-covered or mixed UCSs.

### National patterns and size-specific characteristics of UCSs

**Figure 2.**
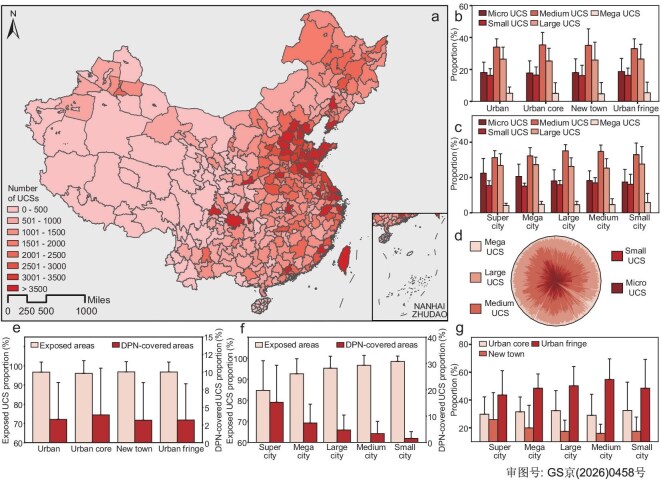
Nationwide distribution and spatial statistics of UCSs. (a) Number and spatial distribution of UCSs. (b) Proportion of UCSs by size within different urban regions. (c) Proportion of UCSs by size in cities of different population scales. As suggested by scholars [84], urban areas are classified into three tiers based on boundary data from 1990, 2000 and 2020: (1) urban core (within the 1990 boundary), (2) urban new towns (expanded between 1990 and 2000) and (3) urban fringe (expanded between 2000 and 2020). Cities are classified by resident population into small (<500 000), medium (500 000–1 million), large (1–5 million), mega cities (5–10 million) and super cities (>10 million) [85]. Micro, Small, Medium, Large and Mega denote very small (<400 m^2^), small (400~1600 m^2^), medium (1600 m^2^~1 ha), large (1~10 ha) and very large UCS (>10 ha), respectively. (d) Composition of UCSs across all prefecture-level cities. The circle is evenly divided into 372 sectors, each representing a prefecture-level city. (e) Proportion of exposed or DPN-covered areas within different urban regions. (f) Proportion of exposed or DPN-covered areas in cities of different population scales. (g) Proportion of UCS number s in different urban regions across cities of different population scales. Table S17 and S18 detail the UCS area and number for each city, respectively. Table S19 presents the national ranking of cities.

Nationwide mapping identified 541 177 UCSs in China in 2020, covering 12 290 km² (Fig.  2a). High-density clusters are concentrated in the Yangtze River Delta, Pearl River Delta and Beijing–Tianjin–Hebei, with inland hotspots in Chongqing and Xi’an. While activity remains the most intense in coastal regions, significant construction is also observed in central and western metropolitan hubs, driven by transport and industrial investment [37]. Across all cities, UCSs are concentrated in urban fringes (50.6% ± 17.5%), reflecting continued outward expansion (Fig.  2b and g). However, 31.4% ± 17.3% of UCSs are already embedded in urban cores (pre-1990 fabric), indicating a renewed focus on inner-city redevelopment. City-size stratification reveals (Fig.  2g) that, in small cities (<0.5 million residents), minimal land-price gradients shift more UCSs toward older urban cores and 1990–2000 expansion belts [38]. Although current policies prioritize renewal, both first- and strong second-tier cities such as Beijing, Wuhan and Chengdu display mixed renewal and expansion patterns.

UCS sizes are consistent across city tiers (Fig.  2b–d). Micro–small UCSs typically represent fine-grained redevelopment within individual block levels, with their density (i.e. number of sites per km^2^) serving as a key indicator of the relationship between urban renewal and the improvement of street-scale livability [39,40]. In contrast, larger-scale UCSs are generally associated with more extensive district-level developments, where the number of sites often reflects broader, long-term urban-renewal strategies, infrastructure development and spatial restructuring [41,42]. In particular, medium UCSs (1600 m²–1 ha) are most common (34.0% ± 5.2%), aligning with typical land parcels under China’s bid–auction–listing system. This scale also suits the full-block renewal of legacy industrial and housing plots [43]. Large UCSs (1–10 ha, 26.5% ± 7.5%) support high-rise clusters and industrial parks, anchoring new-town growth. Their concentration in the fringes of smaller cities reflects shifting land supply and investment patterns. Mega UCSs (>10 ha) are rare but cluster around major urban-growth hubs. Twenty-five cities host >160 such sites (Table S18), sharing traits of high administrative rank, strong population inflows (2015–2020 average > 700 000) [44], robust investment capacity and specialized industries.

**Figure 3.**
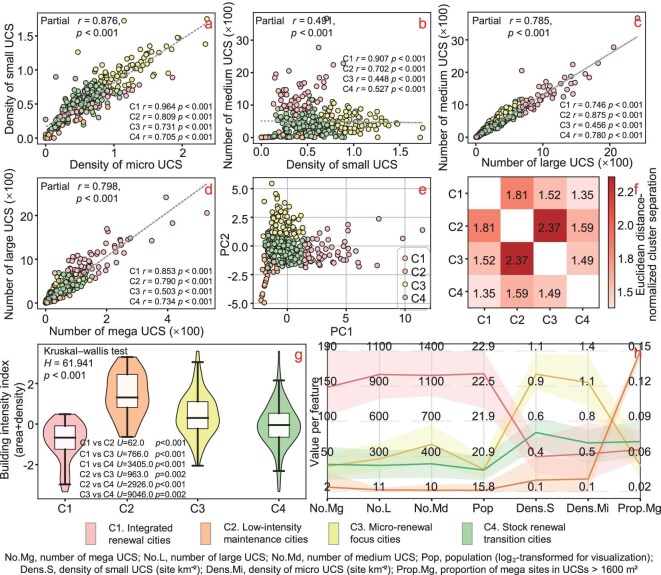
Multiscale coordination and differentiation of UCS sizes. (a–d) Pairwise relationships between UCS size classes with urban area controlled, in which *P* values are Holm-adjusted [86] within families (four tests per full-sample/cluster set): (a) micro-UCS density vs small-UCS density; (b) small-UCS density vs number of medium UCSs; (c) number of medium vs large UCSs; (d) number of large vs mega UCSs. Point markers denote city clusters (C1–C4), consistently across panels. Reported partial correlations are shown in each panel. (e) Visualization of the K-means clustering results in the principal component space derived from the UCS-indicator system. (f) Inter-cluster separation among the four urban-renewal patterns. The heat map reports a separation ratio ${R}_{i,j} = d( {i,j} )/{\mathrm{max}}\{ {{\mathrm{within}}( i ),{\mathrm{\ within}}( j )} \}$, where the numerator is the mean Euclidean distance between all pairs of samples from clusters *i* and *j*, and ‘within’ is the mean pairwise Euclidean distance among samples within each cluster. Values of >1 indicate separation exceeding the internal dispersion of either cluster. (g) Distribution of the building-intensity index across the four urban-renewal patterns. The index integrates the housing construction area and the housing construction density. (h) Cluster-center profiles of the four urban-renewal patterns.

The number of medium and large UCSs is highly correlated (Fig. 3c, partial correlation *r* = 0.785, *P* < 0.001), indicating an entrenched development paradigm that combines block-scale infill with district-scale expansion. In contrast, small (400–1600 m²) and micro (<400 m²) UCSs—comprising 16.7% and 20.5% of the total—are concentrated in residential zones. Micro UCSs function as ‘capillaries’ for pocket parks, elevator retrofits and storm-water nodes; small UCSs host neighborhood centers or compact public facilities. In >70% of the cities, the residential density is ~1.2 ± 0.7 micro and small sites/km², but a long tail reaches 3–4 sites/km², indicating fragmented renewal or informal activity. Their densities are strongly correlated (Fig. 3a, partial correlation *r* = 0.876, *P* < 0.001), underscoring their spatial and functional coupling.

In addition, this study, based on the nationwide inventory, reveals systematic differences in spatial density and environmental disturbance potential across UCSs of varying scales. While traditional urban environmental governance tends to focus on mega UCSs, it shows that micro (<400 m²) and small (400–1600 m²) UCSs are widely distributed. These sites typically involve simple construction organization, short approval processes and short but frequent disturbance cycles, often lacking dedicated regulatory oversight and standardized control measures in most cities [45]. Overlaying UCS distribution with high-resolution nighttime light imagery reveals a strong spatial coupling between UCSs and nocturnal light emissions (Fig. S15), indicating a notable contribution to light pollution. UCS exposure types also follow clear gradients: exposed sites dominate, but DPN coverage declines from core to fringe and with city size (Fig.  2e and f). Higher DPN use in large cities and inner zones reflects stricter environmental controls, while low coverage in small cities and peripheries highlights regulatory gaps. Meanwhile, nationwide DPN coverage is <10%, emphasizing the limited reach of existing dust-proof measures. Using a covariate-matching design with a comprehensive set of geospatial controls, including meteorology, topography, the built environment, transportation intensity, industrial density and population density, we find that construction activities are associated with higher annual mean PM₂.₅ concentrations, whereas the current DPN coverage shows no meaningful reduction in ambient particulate levels (Supplementary Results and Tables S20 and S21).

### Typology of urban-renewal patterns based on UCS size profiles

K-means cluster analysis (Table S22) of UCS size distribution groups China’s 371 prefecture-level cities into four distinct typologies (Fig. 3), each representing a prevailing mode of urban construction activity, excluding Sansha due to the absence of detected UCSs. Figure 3e visualizes the four urban-renewal typologies in the principal component space derived from UCS size indicators. Beyond the overall scaling captured by PC1 (city population and the numbers of medium–mega sites), PC2 distinguishes two contrasting renewal configurations: one characterized by dense micro- and small-site activities and the other dominated by a higher share of mega projects. Notably, cities are distributed along these two directions in parallel rather than along a single continuum, indicating the coexistence of two structurally distinct renewal tracks within the national urban system. Housing construction data, compiled from municipal statistical yearbooks [46,47], validate significant differences both overall and pairwise among the four typologies (Fig. 3g). Further details can be found in the Supplementary Results.

- Low-intensity maintenance cities (29 in total, e.g. Altay, Fangchenggang, Diqing) are in early urbanization (or experiencing slow or even negative growth), marked by low urban population (mean: 319419, Interquartile range (IQR): 38588–452175), limited construction activities (mean total UCSs: 167.17 sites, IQR: 25–234 sites), loose spatial layouts and scattered large projects (mean mega UCSs: 19 sites, IQR: 4–28 sites). Geography and policies jointly drive their urbanization, forming a decentralized pattern of small and medium-sized towns distinct from the eastern market-driven model. Meanwhile, frontier location, ethnic cultural preservation and ecological vulnerability constitute the core constraints for their urban renewal.
- Micro-renewal focus cities (78 cities, e.g. Qiqihar, Taiyuan) are small to medium-sized cities (mean urban population: 1.46 million, IQR: 784175–1.98 million), mainly in central, western and northeastern China. They are former industrial centers facing economic decline, demographic aging and reduced rural in-migration. Their urban renewal is predominantly microscale, as reflected by high densities of micro–small UCSs (mean: 2.21 sites/km², IQR: 1.77–2.51 sites/km^2^). Development is shaped by block-level regeneration, infill and community upgrades. Infrastructure decay, inefficient land use and weak private-sector engagement limit urban development [48,49]. As a result, government-led small-scale interventions have replaced large-scale redevelopment as the dominant mode.
- Stock renewal transition cities (205 in total, e.g. Sanming, Shaoguan, Kaifeng) (mean urban population: 1.42 million, IQR: 671465–1.93  million) constitute the pivotal force of China’s mid- to late-stage urbanization outside major metropolitan hubs. Their UCS density is generally high (mean total UCS density: 3.19 sites/km^2^, IQR: 2.71–3.80 sites/km^2^; mean micro–small-UCS density: 1.07 sites/km², IQR: 0.84–1.35 sites/km²), indicating significant spatial disturbance and a prevalence of ‘repair while in use’ and ‘embedded micro-renewal’ approaches. The low proportion of Mega UCSs among medium-sized and above UCSs (mean: 7.08%, IQR: 5.56%–8.90%) indicates a transition to fine-grained, decentralized development prioritizing community-level enhancements over large-scale demolition, in alignment with the Ministry of Housing and Urban-Rural Development’s policy of ‘refinement over demolition’ [50,51]. Meanwhile, the notable number of large and mega UCSs (mean: 281.49 sites, IQR:134–390 sites) indicates that, while mid-scale functional renewal is underway, some degree of large-scale redevelopment (e.g. old-town regeneration, resettlement housing) continues in certain areas. Overall, these cities are simultaneously undergoing incremental expansion and stock optimization, representing a typical transitional stage from growth-oriented development to stock-oriented renewal.
- Integrated renewal cities (59 cities, e.g. Wuhan, Beijing, Chengdu), most with urbanization rates of >80%, represent China’s first-tier and strong second-tier cities that have entered a phase of intensive construction (mean total UCS density: 2.68 sites/km^2^, IQR: 1.99–3.21 sites/km^2^) and strategic urban renewal. Exhibiting a ‘saturated core–expanding fringe’ development pattern (see Fig. 2g for super and mega cities), their renewal strategies integrate fragmented, high-frequency micro-interventions with large-scale, systemic redevelopment. The distribution of micro–small UCSs (mean: 0.96 sites/km^2^, IQR: 0.53–1.26 sites/km^2^) reflects embedded, small-scale, function-oriented upgrades. Concurrently, a substantial number of UCSs exceeding 10 ha (mean: 169 sites, IQR: 106–203 sites) support district-level regeneration, land restructuring and functional reorganization. Their funding mechanisms are primarily market-driven, complemented by urban planning, forming a tripartite framework of government guidance, enterprise implementation and public participation.

Collectively, these four archetypes illustrate a transitional landscape of urban renewal in China. While large-scale, land-intensive expansion continues to dominate in first-tier and strong second-tier cities, small-scale, quality-driven interventions are gaining strategic importance—especially in larger, more mature urban systems.

### Temporal and cross-sensors evidence

National guidance on urban renewal has been rolled out since 2019, while local implementation has varied across cities. To test temporal robustness, we (i) stratified the model Google Earth test tiles by acquisition season and found stable performance for key UCS classes across seasons (Table S8), although occasional phenology-related confusions (e.g. fallow cropland/low-cover grassland) may still occur; and (ii) added a multi-year Shenzhen case (2021–2024) to illustrate the interannual turnover and parcel-level site trajectories via cross-year parcel linking (Fig. S9). We additionally benchmarked the 2025 Q2 Futian (Shenzhen) outputs against an official construction-site list for external reference (Table S25), achieving parcel-level agreement of >83%. In collaboration with the Shenzhen environmental monitoring agency, the workflow has been piloted to support routine construction-site screening and reporting. For cross-sensor generalization, we further applied the same pipeline, without architectural changes, to five very high-resolution (VHR) remote sensing datasets (Pleiades, GF-7, GF-2, GF-1 and ZY-3) across five cities, achieving consistent accuracy (OA: 89.0%–93.3%; Table S7 and Method 2). Beyond the national 2020 atlas, we retrospectively inferred 2015 for six cases (Table 2) and compared 2015 vs 2020:

**Table 2.** Comparative statistics of UCSs across representative Chinese cities, 2015 vs 2020.

					Number of UCSs					
Typology of urban-renewal patterns (2020)		Year	UCS area (km^2^)	DPN area (km^2^)	Micro	Small	Medium	Large	Mega	Total
Chongqing urban core	Integrated renewal	2015	204.60	0.058	530	378	890	1261	362	3421
		2020	128.27	1.22	458	472	1332	1450	270	3982
										
Chongqing peripheral counties	Stock renewal transition	2015	18.18	6.8e-5	62	37	92	153	31	375
		2020	19.54	0.04	149	122	234	165	39	709
Ordos	Low-intensity maintenance	2015	77.33	0.21	229	116	269	534	186	1334
		2020	31.69	0.54	192	105	202	218	71	788
Yangzhou	Stock renewal transition	2015	41.66	0.042	247	209	761	753	74	2044
		2020	32.51	2.54	636	593	1163	689	43	3124
Beijing	Integrated renewal	2015	299.65	15.36	2390	1431	2950	3453	661	10 885
		2020	199.01	79.38	4516	2623	3669	2414	395	13 617
Qinhuangdao	Micro-renewal focus	2015	45.14	1.01	149	98	339	484	85	1155
		2020	65.31	5.87	514	328	711	536	118	2207

‘Chongqing urban core’ refers to the municipality’s main urban districts; ‘Chongqing peripheral counties’ refers to its outer county jurisdictions. The 2020 typology is a data-driven reference class for 2015–2020 comparison, not a policy designation.

Chongqing (urban core vs peripheral counties): core areas show rising counts but shrinking total areas and fewer mega sites, consistent with stricter land control and the 2020 renewal agenda [52,53]; peripheral belts exhibit rising counts across sizes with roughly stable areas, reflecting multicenter expansion under coordinated development [52].

Ordos: concurrent decline in counts and area, especially large/mega sites, aligning with destocking and shantytown-renovation initiatives [54,55].

Yangzhou: counts increase while total areas fall and the mix shifts from micro/small/medium toward planned block-scale renewal under the 2011–2020 master plan [56] and the 2015–2030 historic-city program [57,58].

Beijing: higher counts, lower total area and greater DPN coverage, consistent with the 2016–2035 City Master Plan (‘control increment, optimize stock’) [59,60].

Qinhuangdao: growth in counts and area, with micro/small/medium increases and selective mega projects under the ‘Urban Double-Repair’ [61] program and community-level micro-updates [62].

Taken together, as a national cross-sectional inventory, the atlas does not resolve complete site life cycles; multi-year updates are needed to quantify turnover and trajectories. These backcasts complement the 2020 nationwide atlas and indicate that our proposed work is both policy-responsive and extensible across years and sensors when multi-temporal VHR imagery is available.

## DISCUSSION

This study provides, for the first time, the nationwide identification and quantification of UCS distributions. Notably, ‘capillary-scale’ updates have been under-identified by prior methods [12,18,63,64]. Our sub-meter deep-learning approach detects UCSs down to 10 m² and successfully recovers the micro and small sites in 2020 (20.5% and 16.7% of all UCSs; Fig. 2d), offering a high-resolution ‘geographic microscope’ for urban spatial change. Beyond revealing previously neglected renewal units, these data provide a quantitative basis for differentiated regulation, resource allocation and day-to-day governance. Overall, urban renewal in China is shifting from linear, single-scale transformations toward a multiscale, nested system with complementary functions and staggered timing. From the micro perspective, UCSs of different sizes do not occur independently, but follow distinct patterns. Medium–large and large–mega pairs are strongly coupled (Fig. 3c and d), supporting district-level renewal such as old-city reconstruction and new-town development. Micro–small co-occurrence within built-up areas forms a high-frequency, ‘capillary’ network that improves street-scale livability and accessibility (Fig. 3a). For the small–medium linkage (Fig. 3b), the updated analysis shows cluster-specific heterogeneity (as tested via likelihood ratio test (LRT) in Table S16): the association is strongest in integrated/systemic renewal cities (C1), evident in low-intensity cities (C2), shallow but positive in micro-renewal cities (C3) and in stock-transition cities (C4). These patterns indicate coordinated yet differentiated multiscale updating, with capillary micro-renewal alongside block- and district-scale projects, recurrent across city sizes. Building on this multiscale organization, the principal component analysis (PCA)-based typology (Fig. 3e) further suggests a dual-track pattern of urban renewal in China. Rather than implying a codified governance regime, we use ‘dual-track’ as an observational descriptor: cities can exhibit renewal dominated by fine-grained, stock-oriented micro/small interventions or renewal accompanied by more project-led, incremental expansion signatures, and the two logics may coexist in different mixes across the urban hierarchy. Since 2019, national guidance on ‘urban renewal’ has prioritized refinement over demolition and the improvement of existing stock [65]; to our knowledge, there is no national directive that explicitly mandates two institutionalized tracks. Within this context, the four data-driven clusters align with two renewal logics: stock-oriented and incremental/transition. Group differences are significant (Fig. 3g) and six city backcasts from 2015 to 2020 show directional shifts in counts, area and size composition that are consistent with the stated policy emphases (Table 2). To bridge spatial observation and policy analysis, we summarize data-derived links between UCS indicators and renewal tracks in Table 3; the long form with quartile thresholds, 95% confidence intervals (CIs) and city-year cases is provided in Table S24 (operational, non-causal). Using this crosswalk, we explicitly interpret the four typologies as different mixtures of the two logics. Stock-oriented signatures are characterized by high micro/small densities and a low mega share, whereas incremental/transition signatures show elevated large/mega activity and strong large–mega coupling; integrated renewal cities typically exhibit hybrid signals, while micro-renewal and stock-transition cities lean more towards being strongly stock-oriented. For example, Huainan (2020) [66,67] aligns with stock-oriented signatures (high micro-UCS density), whereas Xiamen (2020) [68] aligns with incremental/transition signatures (high mega-site share). This mapping transforms raw UCS data into diagnostic tools, enabling authorities to monitor urbanization trajectories—shifting from megacity micro-interventions to provincial capital expansions—with high precision. Our analysis is observational and we do not claim causal effects. We triangulate evidence via: (i) cross-sectional regularities in multiscale coupling and clustering across 371 cities, (ii) external yearbook proxies that differentiate groups overall and pairwise (Table S14 and S15) and (iii) 2015–2020 trajectories that track documented policy priorities. Causal claims are reserved for future work using longitudinal administrative records and quasi-experimental designs, for which we have registered an indicator-to-construct schema (Table 3).

**Table 3.** Operational crosswalk from UCS indicators to renewal tracks and policy emphases (short version).

Indicator	Signal (data-derived)	Cases (refs)	Policy emphasis	Track
Micro-UCS density (sites/km²)	High ≥ Q3(0.826)	Huainan-2020^[s24- Huainan]^, Tiemenguan–2020^[s24- Tiemenguan]^, Beijing–2020^[s24- Beijing]^	Embedded micro-renewal; block upgrades	Stock-oriented
Small-UCS density (sites/km²)	High ≥ Q3(0.733)	Baoting–2020^[s24- Baoting]^, Songyuan–2020^[s24-Songyuan]^ Yangquan–2020^[s24- Yangquan]^	Community upgrades; infill development	Stock-oriented
Medium-UCS count	High ≥ Q3(675)	Jinan–2020^[s24- Jinan]^, Panjin–2020^[s24- Panjin]^	If coupled with micro/small ↑ → Stock; If coupled with Mega↑ → Incremental/transition	Either, context-dependent
Large-UCS count	High ≥ Q3(460)	Wuhan–2020^[s24- Wuhan]^, Changchun–2020^[s24- Changchun]^, Urumqi–2020^[s24- Urumqi]^	District-scale redevelopment; land readjustment; resettlement neighborhoods	Incremental expansion
Mega-UCS count	High ≥ Q3(76)	Xiamen–2020^[s24- Xiamen]^, Changsha–2020^[s24- Changsha]^	New town/park expansion; TOD corridors; industrial platforms	Incremental expansion
Mega share among sites of >1600 m²	Low ≤ Q1(0.049)	Luohe–2020^[s24- Luohe]^, Weinan–2020^[s24- Weinan]^	Destocking; historic-city protection	Stock-oriented
Population of city	High ≥ Q3(2 438 261)	Nanning–2020^[s24- Nanning]^, Luoyang–2020^[s24- Luoyang]^	Population and land-use control	Incremental expansion

Signals are quartiles (values shown); units standardized (sites/km², count/city, ha, %). Thresholds are data-derived (quartiles unless noted) on built-up-area-normalized metrics. Mappings are operational and non-causal. Full thresholds, uncertainty (95% Confidence Interval), decision rules and illustrative case sources are provided in Table S24. The superscripts in the ‘Cases (refs)’ column S24-XX map to the full case descriptions and supporting references in Table S24. TOD corridors refer to transit-oriented development corridors—areas along high-capacity public transit where denser, mixed-use, walkable development is encouraged around stations.

Based on microscale observations of construction activity, the dual-track pattern emerges across diverse urban contexts [65]. Rather than a single ‘growth–decline–renewal’ sequence [69–71], Chinese cities exhibit a mosaic in which expansion and stock optimization coexist, with the mix conditioned by development stage and governance capacity. As national priorities pivot from quantity-driven growth to quality-oriented development [72], community-scale, context-specific renewal has gained prominence and stock-transition cities constitute a large share of the urban landscape (205 of 371 prefecture-level cities). Whether this pattern can deliver spatial equity across city types, from frontier towns to super/mega cities, requires long-term empirical tracking and systematic policy evaluation. Unlike Western settings in which private capital often dominates, China’s urban transformation is shaped by state-mediated adaptive governance [73,74]. Our 4-fold typology reflects this diversity: low-intensity maintenance cities depend more on public investment to offset ecological and geographic constraints, whereas integrated renewal cities combine limited outward expansion with dense micro-interventions. These differences underscore that urban renewal is not uniform, but context-sensitive, driven by life-cycle stage, governance capacity and spatial conditions. National frameworks on ‘urban renewal’, ‘old-city renovation’ and ‘shantytown renovation’ provide a baseline, yet local implementation must balance standardized guidance with site-specific adaptation. For example, ecologically fragile frontier cities may prioritize ecological compensation before large-scale development, while traditional industrial hubs benefit from granular, block-level interventions. Continued optimization of planning frameworks to address regional disparities remains an important direction for research and practice, together with timely evaluation of implementation outcomes. In sum, the high-resolution UCS mapping developed here provides a robust data foundation for recognizing renewal scales and matching instruments to contexts. Urban-renewal policies should move from uniform prescriptions toward targeted and precise governance, addressing the distinct needs of each city and region [75,76]. By building a shared structural understanding, stakeholders can align long-term planning of renewal paths, urban functions and spatial structure, advancing urban renewal toward more efficient and sustainable development.

## MATERIALS AND METHODS

### Semi-automatic UCS-sample collection

We developed a semi-automatic annotation framework integrating human–machine interaction with weakly and semi-supervised learning to detect pixel-wise high-confidence exposed areas, DPN areas, buildings and background (Fig. S1). An image classification network (RegNetY-4.0GF [77]) was first trained by using image-level labels to identify candidate UCS images nationwide, which were subsequently manually verified, yielding 163 000 samples (Im-UCS). Pixel-wise annotations were then generated by using UniMatch [78] and BldgNet [79], refined by the Segment Anything Model [80], and further optimized through a fusion strategy to resolve label conflicts.

### UCS-detection model

The proposed UCS-detection model adopts a dual-branch encoder–decoder architecture with a feature merge module and dual segmentation heads (Fig. S5). Each branch employs MixTransformer (mit_b1) [81] as the encoder. One branch extracts spectral–spatial features from multispectral inputs, while the other focuses on spatial layout and texture by using the panchromatic band. A Bottleneck Attention Module is introduced into the merge module to adaptively fuse spectral–spatial features via channel and spatial attention mechanisms.

### Validation of STTBNet model and the nationwide UCS map

As seen in Table 1, the STTBNet demonstrated robust performance across all categories. Due to interference from complex backgrounds (e.g. irregularly placed construction materials, equipment and tools on the site), the pixel-level accuracy for exposed surfaces was slightly lower compared with those of other categories. Meanwhile, the sensitivity of the pixel-wise exposed areas still exceeded 80%. The nationwide UCS map achieved an overall accuracy of 94.3%, indicating that the developed national-scale product is highly reliable and suitable for practical applications.

### Clustering K-means cluster analysis of UCS size distributions

China’s urban-renewal system exhibits a notable pattern of coexistence between multiscale collaboration and differentiated development. To systematically analyse the variations in urban renewal across cities at different spatial scales, we constructed a 3D indicator system encompassing the ‘micro–meso–macro’ levels. At the micro level, we selected the density of the micro and small UCSs; at the meso scale, the number of medium UCSs was used to reflect the intensity of the neighborhood-scale redevelopment; and, at the macro level, the number of large and mega UCSs was included to indicate the urban-development intensity, and the proportion of mega UCSs among all UCSs of >1600 m² was introduced to reveal the dominance of large-scale projects. In addition, city population was included as a key variable to comprehensively assess the influence of city size on the renewal patterns. Population data were sourced from a 100-meter-resolution gridded population dataset, which integrates China’s Seventh Census and Big Geospatial Data, demonstrating high accuracy (correlation coefficient of 0.8936) [82]. The city population was estimated and detailed as follows:


(1) \begin{eqnarray*}
{\mathrm{Population}} = {\mathrm{\ }}\mathop \sum \limits_{i = 0}^N \textit{Pixel}_i,
\end{eqnarray*}

where *N* denotes the total number of pixels within the city boundary and $Pixe{l}_i$ represents the population value of the *i*-th pixel.

Based on this indicator system, we applied the K-means clustering algorithm [83] to conduct a multidimensional analysis of 371 prefecture-level cities across China, ultimately identifying four distinct urban-renewal patterns with significant differences.

## Supplementary Material

nwag141_Supplemental_Files

## Data Availability

The data and resources underlying this article are available in the following repositories: Source Code: the source code for the entire pipeline is available in GitHub at https://github.com/wangqiaoqiaoqiao/UCS. Datasets: the underlying datasets, including manually delineated labels and metadata for reconstruction, are available in Zenodo at https://dx.doi.org/10.5281/zenodo.17404901. Online demo: an online demo for model validation is accessible at https://irsip.whu.edu.cn/wwr_test/index_UCS.php.
