# Supplementary material for: Deep-learning-based sub-meter urban construction-site mapping reveals China’s dual-track urban renewal
Source: Natl Sci Rev. 2026 Mar 10;13(7):nwag141. doi: 10.1093/nsr/nwag141 (PMC13105178; doi:10.1093/nsr/nwag141)
Supplement: nwag141_Supplemental_Files [file nwag141_supplemental_files.zip › Supplementary material_v2_clean.docx]

Supplementary Materials for

**Deep-learning-based sub-meter urban construction site mapping reveals China's dual-track urban renewal**

Jiayi Li et al.

*Corresponding author: Xin Huang and Liangpei Zhang. Email: [xhuang@whu.edu.cn](mailto:xhuang@whu.edu.cn); zlp62@whu.edu.cn

## Supplementary Method 1: Sample collection

### **SM1.1 Data sources for nationwide urban construction sites (UCS) mapping**

Initial dataset for training the sample collection method: previous studies [S1,2] have manually labeled 36,000 samples, each consisting of a 1-m true color satellite image sized 512×512, pixelwise annotations, and an image-level label (i.e., indicating whether UCS region larger than 100m×100m). The categories of pixel-level annotation include DPN-covered areas, exposed areas, background. According to the image-level annotations, these 36,000 samples are categorized into 3000 DPN-covered UCS samples, 3000 exposed UCS samples, and 30,000 background samples.

For nationwide sample collection and model prediction: true-color satellite images with a resolution of 0.5 meters were collected from Google Earth. These images, acquired between 2019 and 2020, encompass urban areas in China as delineated by the widely used Global Urban Boundary (GUB)[S3] and its improved version, GUSV[S4]. To facilitate deep learning, these Google Earth images were cropped into 1024×1024 pixel tiles with a 1/16 overlap, yielding 3,416,880 images. Among these, at least two tiles (1024×1024 pixels) from each province were randomly selected and manually annotated for accuracy validation. Annotation was performed by three experienced interpreters via independent labeling and adjudication. To avoid patch-boundary bias, nationwide 512 × 512 tiles were interpreted within their spatial context to ensure categorical continuity. Google Earth imagery usage complies with non-commercial scientific research guidelines; all such figures are credited “Image data © Google.” Ultimately, 1000 annotated images were employed to assess the accuracy of the deep learning model and the nationwide UCS product.

### **SM1.2 Semi-automatic UCS sample collection**

UCS mainly includes exposed areas, temporary buildings, DPN-covered areas, and construction facility storage, typically adjacent to buildings. Thus, the proposed UCS detection method relies on semantic segmentation, which assigns semantic labels (e.g., buildings, exposed areas, DPN-covered areas) to each pixel. This process heavily relies on a lot of fine-grained annotated samples, making it extremely time-consuming and labor-intensive [S5]. This significantly limits the implementation of nationwide UCS detection tasks. Inspired by Segment Anything (SAM)[S6], high-quality segmentation results can be achieved by manually annotating a portion of pixel-level samples (e.g., SAM collected 4.3M masks from 120k images to form an initial dataset ) and building a semi-supervised based data engine to scale up annotations. However, China's vast size and complex terrain make it difficult to obtain enough initial pixel-level annotations. To address this, a semi-automatic sample annotation approach that combines human-machine interaction with weakly-supervised and semi-supervised learning is developed (Supplementary Fig.1). This approach enables the acquisition of pixel-level UCS annotations for massive unlabeled samples, even with only a small number of image-level annotations.

Specifically, 36,000 training samples with image-level annotations are used to train an image classification deep network RegNetY-4.0GF[S7] to identify candidate UCS images from nationwide Google Earth images. RegNetY-4.0GF is regarded as an efficient network with satisfactory classification accuracy for UCS detection[S1,2] RegNetY-4.0GF consists of three components: a stem (which includes a 3 × 3 convolution layer with a stride of two), a network body dedicated to computation, and the head (which employs global average pooling followed by fully connected layers) to output the prediction. The candidate images are then manually verified, and 163k UCS images are retained (called as Im-UCS). Next, for each image in Im-UCS, we first perform pixel-level annotation for each UCS-related element (i.e., buildings, exposed areas, DPN-covered areas), and then resolve conflicting labels using a fusion strategy.

For pixel-wise exposed areas sample collection, a semi-supervised sematic segmentation, UniMatch[S8] (see supplementary method 1 for more details), was trained using 3000 labeled exposed UCS samples and 163k unlabeled Im-UCS images. In this article, UniMatch use RegNetY-4.0GF as the encoder backbone and the original U-Net as the decoder. During training, UniMatch classifies each pixel in Im-UCS as either exposed areas or background. Only pixels with a confidence greater than 0.95 are classified as exposed areas or background, while the remaining pixels are labeled as uncertain. Then, SAM[S6] leverages its deep learning model to extend and refine the segmentation across each Im-UCS image based on current semi-supervised annotation, and predicts pixel-level labels accordingly. The pixel-wise DPN-covered areas sample collection are conducted in a similar manner. Pre-trained sematic segmentation network BldgNet (see supplementary method 1 for more details)[S9] is employed to generate building labels on Im-UCS images. Pixels obtained from the first UniMatch with a confidence greater than 0.999 in the background category are considered to be the final background pixels. For pixels with conflicting identifications (*e.g.*, identified both as DPN-covered areas and exposed areas), the labels are assigned based on the following priority hierarchy: buildings have the highest priority, followed by DPN-covered areas, then exposed areas, and finally, background. Except for the regions labeled as DPN-covered areas, exposed areas, buildings, and background, the remaining pixels are considered uncertain.

According to above semi-automatic approach, we have labeled a total of 163k images sized 1024×1024, with label types including: DPN-covered areas and exposed areas, buildings, background, and uncertain.

[Supplementary Fig. 1]

### **SM1.3 UniMatch (Unified Dual-Stream Perturbations approach)**

UniMatch[S8] is a semi-supervised semantic segmentation method designed to guide the learning of a large amount of unlabeled data using a small number of labeled samples, as illustrated in Supplementary Fig. 2. For labeled images, UniMatch computes the loss function between the predictions and the labels. For unlabeled images, UniMatch ensures that the model produces consistent predictions across different perturbed versions of the same image. To achieve this, it uses the model's predictions on weakly perturbed images as pseudo-labels to guide and supervise the predictions for other, more strongly perturbed versions. Here, weak perturbation refers to simple data augmentation operations on unlabeled images, such as rotation and flipping. Strong perturbation involves more complex data augmentation operations on unlabeled samples, such as color jittering, blur and grayscale. Different augmentation operations on the same image will result in various perturbed versions. UniMatch has demonstrated outstanding performance across multiple datasets, including natural and remote sensing images[S8], validating its efficacy as a benchmark solution for semi-supervised segmentation. Therefore, in our study, we adopted UniMatch as the primary technique for semi-supervised learning in our semi-automatic sample annotation approach to generate pseudo-labels for images in Im-UCS.

[Supplementary Fig. 2]

### **SM1.4 BldgNet (Building Extraction Network)**

BldgNet[9] constitutes a Transformer-driven neural framework optimized for extracting buildings on a large scale. It effectively mitigates three major challenges that are commonly encountered in large-scale building extraction, including scale diversity, boundary precision, and class imbalance[S9–11]. Scale diversity refers to the varying sizes of buildings, ranging from small structures to large complexes, making it difficult for models to comprehensively capture features across all scales. Boundary precision highlights the issue where, due to down-sampling operations in deep learning models, detailed information of buildings is easily lost, leading to difficulties in accurately delineating boundaries. Class imbalance highlights that buildings occupy less than 10% of the total land surface[S9,12]. Consequently, compared to the abundant background features, there is an uneven distribution of samples during the building extraction process, with relatively fewer building samples and a predominant number of background samples. By optimizing these issues, BldgNet achieves a strong balance between accuracy and efficiency, delivering highly satisfactory results[S9]. Therefore, in our study, we used the well-trained BldgNet network to generate building pseudo-labels for the images in Im-UCS.

### **SM1.5 Training details**

RegNetY-4.0GF for image classification: A dropout layer (rate: 0.2) was added before the fully connected layer to prevent overfitting. The model was optimized using the SGD optimizer with an initial learning rate of 0.001 and trained for 10 epochs.

UniMatch for pixel-wise DPN-Covered area sample collection: Semi-supervised learning was conducted with a labeled-to-unlabeled sample ratio of 1:8, using the Adam optimizer with a fixed learning rate of 0.001.

UniMatch for pixel-wise exposed area sample collection: Semi-supervised learning was conducted with a labeled-to-unlabeled sample ratio of 1:8, optimized using the SGD optimizer with Nesterov momentum. The initial learning rate of 0.001 was dynamically adjusted using a cosine schedule. Training ran for 40 epochs: the first 20 used 3,000 manually annotated exposed UCS samples as labeled samples, while the subsequent 20 incorporated additional manually verified pseudo-samples identified by the current model.

### **SM1.6 Sample category selection**

Urban Construction Sites (UCSs) refer to areas specifically designated for building construction[S13]. During the construction process of UCS, earthworks such as excavation and backfilling are typically carried out[S14,15], which distinguishes the land within UCS from other undeveloped or natural areas, resulting a bare surface with construction traces (referred to as exposed areas). Exposed areas are one of the main sources of urban dust[S15]. To mitigate dust generation during construction, the government mandates that UCS be covered with green plastic dust-proof nets (referred to as DPN-covered areas)[S1,16,17]. By focusing on exposed areas and DPN-covered areas, we can effectively identify UCS under construction.

Buildings typically originate from UCS (as shown in Supplementary Fig. 3 a-b) or are often spatially co-located with UCS (as shown in Supplementary Fig. 3 c-d). In some cases, buildings may also display spectral characteristics similar to exposed areas. Additionally, during the construction process, temporary buildings may appear on UCS (as shown in Supplementary Fig. 3 e-f). To address this issue, we treat buildings as a separate category during UCS extraction training, allowing the model to effectively differentiate between buildings and exposed areas. Therefore, for pixel-level semantic segmentation, we treat exposed areas, DPN-covered areas, and buildings as the three categories that the model needs to extract.

[Supplementary Fig. 3]

In addition to exposed areas, DPN-covered areas, and buildings, UCS also contains ground objects such as construction facility storage, construction waste, and buildings under construction. Considering that these ground objects are relatively scattered and usually located within the interior of UCS, hole-filling is used in morphological post-processing to ensure the integrity of UCS.

### **SM1.7 The result of semi-automatic UCS sample collection**

Supplementary Fig. 4 profiles the UCS sample set from spatial coverage to category composition and local annotations. First, samples span major urban corridors (Bohai Rim–Yangtze River Delta–Pearl River Delta) and inland/border cities, ensuring broad national representativeness. The proportions across all categories are approximately balanced (without background dominance), mitigating class-imbalance risks. From panels c–k, the dataset exhibits diverse appearances with clear textural separation between DPN (fine mesh) and exposed sites (coarse granularity), mixed sites showing multi-material, fragmented boundaries, and high-fidelity pixel-wise annotations that capture boundaries and small structures for fine-grained segmentation. In concert with the Error analysis and Uncertainty quantification. (see Supplementary Method 2), these properties provide effective learning signals for texture/material discrimination and for challenging boundary/small-object cases, thereby supporting downstream accuracy evaluation and typology analysis.

[Supplementary Fig. 4]

## Supplementary Method 2: UCS detection model

### **SM2.1 UCS detection model**

Spectral characteristic from satellite image is one of the most important information for land-cover extraction. Spectral information from satellite imagery is crucial for land-cover extraction[S18] . For example, DPN is a type of green plastic mesh (e.g., Supplementary Fig. 4c and g). Exposed areas and buildings, however, are color-insensitive. Regions with sparse grass, fallow cropland, and unused surfaces can exhibit similar spectral characteristics to exposed construction sites, leading to false alarms when relying solely on spectral data. Excavation activities at UCS typically result in uneven terrain, which creates distinct textural features in satellite images (e.g., Supplementary Fig.3). Therefore, using a semi-automatic dataset with pixel-level annotations, this study develops a spectral-textural two-branch semantic segmentation network for nationwide UCS detection.

The proposed two-branch network consists of two encoders-decoders structures, a merge module, and two sematic segmentation heads. Each auto-encoder employs MixTransformer (mit_b1)[S19] as the encoder and the original decoder, combining efficient hierarchical feature extraction and mixed-scale attention for a balance of performance and computational cost. As seen in Supplementary Fig.5, the first auto-encoder leverages the Transformer architecture structure to capture spectral-spatial information. The second encoder, which takes the panchromatic band as input, focuses on spatial layout and texture, disregarding spectral data. The merge module then combines the spectral-spatial features from the first branch with the textural features from the second. The merge module employs a Bottleneck Attention Module to extract channel (the upper branch in Supplementary Fig.5 and spatial attention (the lower branch in Supplementary Fig.5) from the panchromatic input, which is used as weights to adjust the spectral-spatial features. During training, the first cross-entropy loss (loss 1 in Supplementary Fig.5) optimizes the background, DPN areas, and building pixels, while the second loss (loss 2 in Supplementary Fig.5) targets background and exposed areas. Two losses are equally added. In the prediction phase, the lower decoder is used for background and exposed areas, while the upper branch handles DPN and building predictions.

[Supplementary Fig. 5]

### **SM2.2 Training details**

Manually annotated and semi-automatically collected samples were combined and split into training and validation datasets (9:1 ratio). The model was trained for 40 epochs using the Adam optimizer with a fixed learning rate of 0.001, with independent optimization of each branch in the first 20 epochs and joint training of the entire network in the next 20 epochs.

### **SM2.3 Evaluation metric for model comparison**

To assess the model’s ability to detect and identify UCS, Overall Accuracy (OA), Precision and Sensitivity, the commonly used semantic segmentation metrics in the computer vision field, are utilized in this study[S18,20–22]. OA calculates the proportion of correctly classified samples out of the total, offering a comprehensive measure of the model's performance. Precision assesses the accuracy of the model's predictions, indicating the proportion of samples predicted as a specific category that actually belong to it. Higher Precision corresponds to a lower false alarm rate. Sensitivity measures the model's recall ability, reflecting how effectively it identifies samples that truly belong to a given category. The higher the Sensitivity, the lower the missed detection rate.

### **SM2.4 Test Sample**

As shown in Supplementary Fig. 6, we conducted the delineation of test samples across the entire country. The test samples are divided into model test samples and nationwide map test samples, with each category being selected independently. During the delineation process for each category, we ensured that each of the 34 provinces and municipalities directly under the central government contained at least one test sample. Considering that UCS is primarily concentrated in economically developed and urbanized regions, more test samples were selected from the eastern and southern regions compared to the western and northern regions.

[Supplementary Fig. 6]

To validate the proposed work, 1,000 images (0.5 km × 0.5 km) were stratified, randomly sampled, and manually annotated nationwide. Of these, 500 were labeled at the pixel level (DPN-covered, exposed, building, and background) to evaluate STTBNet, while 500 were object-level labeled (DPN-covered UCS, exposed UCS, and mixed UCS) to assess the nationwide UCS map. The evaluation metrics included Precision, Sensitivity, and Overall Accuracy (OA)[S2]. Sensitivity, defined as the proportion of correct predictions among all manual annotations, highlights the effectiveness of identifying positive cases. Precision measures the proportion of correctly identified results among all predictions, reflecting the accuracy and reliability of positive classifications. OA evaluates the proportion of correctly classified samples across all annotations, indicating overall performance.

### **SM2.5 Method comparison**

We validate the effectiveness of our proposed spectral-textural two-branch semantic segmentation network (STTBNet) by comparing its performance with other models. We compared the following deep learning models, including a multi-scale deformable convolutional neural network (MDCNN)[S17], high-resolution network (HRNet)[S23], position aware circular convolution (ParC-Net)[S24], and token pyramid transformer (TopFormer)[S25]. Among them, MDCNN is a deep learning network designed for green plastic cover mapping, addressing the issues of varying scales and shapes of green plastic covers in complex urban landscapes. Based on MDCNN, researchers successfully achieved accurate identification of green plastic cover areas in Jinan. HRNet is a deep learning network suitable for handling dense prediction tasks. Traditional deep learning models often reduce the image resolution through downsampling to reduce computation, but this can result in the loss of important details. In contrast, HRNet maintains high resolution throughout the process, ensuring that key details in the image are preserved. ParC-Net integrates the advantages of traditional convolution-based models and transformer-style vision architectures, enabling the extraction of global context via convolution operations. With fewer parameters and faster inference speed, it achieves better performance than both lightweight CNNs and Vision Transformers. TopFormer is a lightweight ViT tailored for mobile devices and optimized for tasks that require dense predictions, such as semantic segmentation. The mobile-friendly means it can perform pixel-level dense prediction tasks on mobile devices, such as smartphones. This network strikes a good balance between accuracy and efficiency.

All experiments were conducted on a 64-bit Windows workstation with four NVIDIA GeForce RTX 2080 Ti GPUs (11 GB each), dual Intel Xeon Silver 4210 CPUs (2.20 GHz; 20 physical cores/40 threads total), and 256 GB RAM, using Python 3.7.16, PyTorch 1.10.0+cu102, and CUDA 10.2. Training STTBNet on 163k Google 0.5 m tiles takes ~4.5–5 hours per epoch on four GPUs (~18–20 GPU-hours/epoch), with ~8–10.5 GB GPU memory per card. For inference, the measured throughput is 36.59 FPS on 512×512 tiles at 0.5 m; since each tile covers 0.0655 km², processing 100 km² requires ~1,500 tiles and ~40–45 seconds of pure model inference (≈0.01 GPU-hours per 100 km²), excluding I/O and pre/post-processing. We also report FPS, parameter counts, and FLOPs for STTBNet and baselines in Supplementary Table 1.

[Supplementary Table 1]

We randomly selected 10% (14,670 images for training and 1,630 images for validation) from the Im-UCS dataset to train the models. Each model was trained for 40 epochs with the same parameter settings (Adam optimizer with a fixed learning rate of 0.001). The accuracy on 500 test samples (shown in blue points in Supplementary Fig. 6) is presented in Supplementary Table 2 and the visualization results is shown in Supplementary Fig. 7. The results show that our STTBNet can extract foreground objects more comprehensively while exhibiting fewer misclassification instances.

[Supplementary Table 2]

[Supplementary Fig. 7]

### **SM2.6 Experimental results on model**

We conducted an ablation study on the merge module in STTBNet. Excluding the merge module means training the model using only the spectral spatial features from the first branch of STTBNet. The results in Supplementary Table 3 show that incorporating the merge module enhances the overall performance of the model, reducing the misclassification of exposed areas and the omission of DNP-covered areas, while also improving the extraction of buildings.

[Supplementary Table 3]

To analyze the contribution of each branch, we added a loss-weighting ablation by varying the relative weights between the two losses, covering moderate settings (e.g., 1:2, 2:1, 1:3, 3:1) as well as more skewed settings (10:1, 1:10) and extreme settings (100:1, 1:100). As summarized in Supplementary Table 4, the equal-weighted setting (1:1) provides the most balanced performance across the two UCS-related classes, maintaining strong sensitivity and precision for both exposed areas and DPN-covered areas. In contrast, heavily skewed weights tend to bias the optimization toward one branch and lead to noticeable trade-offs, especially under the extreme ratios. Overall, these results support 1:1 as a robust default that avoids over-emphasizing either loss while preserving balanced class-wise behavior.

[Supplementary Table 4]

We furtherly expanded the ablation study by adding additional comparisons with common feature-fusion and attention modules, including a simple two-branch feature summation (denoted as “baseline”), CBAM, SE block, and CrissCross Attention. The results are reported in Supplementary Table 3, where “×” denotes the variant without any fusion module. As shown, introducing our merge module improves OA from 91.3% (no merge) to 92.1%, and consistently increases both sensitivity and precision for the two key UCS-related classes (exposed areas and DPN-covered areas). Compared with CBAM, SE, and CCA, our merge module achieves the highest OA and yields a more balanced sensitivity-precision trade-off across classes, while maintaining competitive performance for buildings and background. These results support the effectiveness and robustness of the proposed merge design within STTBNet.

A stratified evaluation was conducted on the 500 tiles sampled across 34 provincial-level regions (Supplementary Table 5). Across city-size tiers, OA ≈92–95% was observed, indicating scale-robust performance. Background remained consistently high; Building showed high precision with minor variance; DPN-covered exhibited very high precision with modest sensitivity fluctuation; Exposed areas showed slightly lower sensitivity in mega/super-cities, attributable to denser fabrics and boundary complexity. Overall robustness across scales is supported.

[Supplementary Table 5]

A stratified evaluation was conducted on the 500 tiles sampled across 34 provincial-level regions (Supplementary Table 6). Across zones, OA ≈92.5–93.7% was maintained. Exposed-area sensitivity was lowest in urban cores and higher in new towns/fringes; DPN-covered, Building, and Background remained stable and high. The results indicate good generalization to peri-urban/less-developed contexts.

[Supplementary Table 6]

### **SM2.7 Transferability beyond Google Earth Imagery**

To demonstrate the portability of our framework beyond Google Earth imagery, we expanded our validation to five cities representing diverse geographic locations and urban scales: Zhuhai (southeast coastal, large city), Shenyang (northeast industrial, mega city), Xi’an (northwest, mega city), and the super cities of Beijing and Shenzhen. This evaluation utilizes five different sensors (Pleiades, GF-7, GF-2, GF-1, and ZY-3) with resolutions from 0.5 m to 2.1 m. It is noted that we use only RGB bands and do not apply explicit cross-sensor radiometric/spectral harmonization or domain-adaptation. Instead, images from each sensor are normalized separately with per-band z-score standardization (mean/SD computed from each sensor’s training set). Because the multi-sensor sources share the same broad visible-band modality, the remaining cross-sensor appearance differences are relatively limited for this task and are further accommodated by the cross-sensor replication pipeline. Representative qualitative results in Beijing are provided in Supplementary Fig. 8.

As summarized in Supplementary Table 7, the proposed work achieves consistent high performance across all domestic sensors, with Overall Accuracy (OA) ranging from 89.0% to 93.3%. Notably, the model maintains high precision for complex categories like “Exposed areas” even as resolution decreases to 2.1 m, demonstrating its strong adaptability to varied sensor characteristics. These results confirm that the proposed pipeline is not tied to a specific commercial data provider but can be reliably reproduced using policy-compliant, domestically available satellite data. These demonstrate cross-sensor generalizability to non–Google Earth imagery without architectural changes, and support applicability to other providers and regions where comparable very-high-resolution data are available.

[Supplementary Fig. 8]

[Supplementary Table 7]

### **SM2.8 A multi-year case to illustrate inter-annual turnover**

To demonstrate that the same pipeline can capture multi-year dynamics when repeated imagery is available, we added a Shenzhen case study (2021–2024): we generated annual UCS maps and linked spatially overlapping/adjacent parcels across years to summarize site transitions (e.g., newly started, ongoing, and completed), revealing coherent year-to-year evolution rather than random fluctuations (Supplementary Fig. 9). In collaboration with colleagues from the Shenzhen environmental monitoring agency, this workflow has also been piloted to support construction-site screening and reporting.

[Supplementary Fig. 9]

### **SM2.9 Error analysis and uncertainty quantification on the national UCS map**

(1) Error analysis. Typical failure modes are documented with representative tiles and masks (Supplementary Fig. 10): (i) confusion between exposed construction surfaces and natural/pre-existing bare soils (e.g., low-cover grassland, fallow cropland, tidal flats, see Supplementary Fig. 10a and 10d). Using timestamped Google Earth imagery, we stratified the 500-tile test set by season and found no significant seasonal differences in sensitivity/precision for Exposed and DPN classes (Kruskal-Wallis[S26]; Supplementary Table 8, all *p* > 0.05), indicating that acquisition-date inconsistency is unlikely to be a systematic driver of errors, though isolated confusions (e.g., fallow cropland or low-stature vegetation) can still occur; (ii) confusion between polymer/artificial surfaces (e.g., running tracks, artificial turf, playground plastics, see Supplementary Fig. 10b) and green dust-proof nets (DPN). Since running-track facilities are commonly tagged in open maps, we optionally leverage open POI/venue layers (e.g., AMap/Gaode records of track-and-field grounds) as a lightweight post-processing filter to suppress these false alarms; (iii) missed or boundary-confused detections for very small or fragmented DPN patches (see Supplementary Fig. 10c, 10e and 10f); and (iv) boundary complexity for slender micro-buildings and light/white roofs (see Supplementary Fig. 10a, 10e and 10f). For cases (iii)–(iv), we clarify that parcel buffering with hole-filling is an operational aggregation step: it determines whether nearby fragments are merged into a single UCS parcel and therefore affects parcel counts and size composition. A buffer-radius sensitivity analysis on parcel counts by size (Supplementary Table 9) and pixel-level accuracy on the 500-tile nationwide test set (Supplementary Table 10) indicates that a 25 m buffer is a robust default, balancing the removal of spurious micro-fragments against over-merging.

[Supplementary Fig. 10]

[Supplementary Table 8]

[Supplementary Table 9]

[Supplementary Table 10]

(2) Uncertainty quantification. A Monte-Carlo dropout protocol (10 stochastic forward passes per image) has been implemented on the 500-tile test set. Pixel-level uncertainty is defined by the variance of predictive probabilities; image-level uncertainty is computed as the mean pixel-wise variance (Supplementary Fig. 11). Distributions of image-level uncertainty (overall and per class) and class-conditioned summaries are provided to contextualize reliability. As the typology is computed from city-level aggregates, the influence of isolated pixel-level noise is attenuated.

Taken together, the class-wise sensitivity/precision and OA indicate balanced, high performance across categories, and overall reliability sufficient for nationwide-scale application. From the uncertainty figures (Supplementary Fig. 11-12), overall predictive uncertainty is low-to-moderate and spatially concentrated along class boundaries (exposed vs. DPN) and in micro/fragmented objects, with limited influence on city-level aggregates and the derived typology.

[Supplementary Fig. 11]

[Supplementary Fig. 12]

(3) Non-urban negative controls. We add two negative-control checks in Shenzhen showing the pipeline does not spuriously inflate micro/small UCS densities: (i) Since March 1, 2021, urban renewal in Shenzhen has been governed by the Shenzhen Special Economic Zone Urban Renewal Ordinance[S27] and the Ministry of Housing and Urban–Rural Development’s August 2021 notice on preventing large-scale demolition and construction[S28]. Under this framework, no substantive institutional changes occurred before April 2024 [S29,30], making 2022–2023 a period of relative policy stability. During this interval, the model produces temporally consistent UCS outputs: total UCS area decreases from 46.55 to 41.48 km², while the number of parcels increases from 1,755 to 1,977 (Supplementary Table 11), indicating a policy-driven shift from large-scale redevelopment toward smaller, more fine-grained construction activities; and (ii) in a predominantly non-urban control (Wutongshan scenic area), predicted UCS is essentially zero (0.0041 km²/50.94 km² = 0.00805%), while the adjacent urban region shows a very small ratio (0.050 km²/51.07 km² = 0.0979%). Manual inspection (Supplementary Fig. 13) supports that false positives on non-urban tiles are not systematic.

[Supplementary Fig. 13]

[Supplementary Table 11]

### **SM2.10 Category Selection of UCS**

Construction dust is a significant source of PM2.5 and PM10 emissions[S31–33], and high DPN coverage can effectively reduce construction dust[S1]. To assess the implementation of environmental measures during UCS construction in different cities, after completing the sub-meter level UCS extraction task, we classify UCSs based on the DPN coverage as follows: Exposed UCSs (DPN coverage ratio of 0), Mixed UCSs (DPN coverage ratio greater than 0 and less than 100%), and DPN-covered UCSs (DPN coverage ratio of 100%)[S34].

## Supplementary Method 3: Post-processing for UCS mapping

After inferring the Im-UCS dataset, the results are merged by city, with union processing applied to overlapping regions to reduce omissions. CLCD products[S35] are then used to mitigate false alarms. Specifically, areas such as farmlands, forests, shrubs, grasslands, snow, ice, barren lands, and wetlands, which showed no land cover changes from 2021-2023, are used to exclude exposed areas and DPN within these regions. Similarly, water bodies with no land cover changes during this period are used to filter out intersecting exposed areas and DPN areas.

Morphological post-processing: After filtering, fragmented exposed areas and DPN areas smaller than 10m² are removed. A 25-m buffer zone is applied to merge the remaining exposed areas and DNP areas into a UCS parcel. The rationale behind the selection of the buffer zone radius is evaluated in Supplementary Discussion. Finally, hole-filling is performed to produce the final sub-meter resolution UCS for China.

## Supplementary Results

### **SR.1 Covariate matching approach to reveal the UCS contributions to PMs**

To assess the environmental impact of construction sites, we adopted a covariate matching approach following a recent study[S36]. Annual mean PM₂.₅[S37] and PM₁₀[S38] concentrations in 2020 (1 km resolution) were used as indicators of ambient air quality. The country was divided into 1 km × 1 km grid, and the proportion of construction site area was calculated for each grid. Grids with construction coverage >1% were defined as **UCS grids**, and those with <0.01% as **Non-UCS grids**. To ensure that construction activities occurred only during the study period, we visually confirmed that construction coverage in both UCS and Non-UCS grids was <0.01% in 2024. This yielded 3,403 UCS grids and 11,892 Non-UCS grids nationwide.

Confounding factors include: (1) meteorology (temperature, humidity, wind speed); (2) topography (Copernicus DEM); (3) built environment (impervious surface density); (4) transportation intensity (PCA-summarized OSM traffic and road/rail network densities); (5) industrial density; and (6) population density. Meteorological variables (temperature, humidity, wind speed) are sourced from the ChinaMet dataset, a high-resolution (1 km), long-term multivariate meteorological forcing product for China[S39]; impervious surface area (human activity) derived from the Global ISA 10m dataset (GISA-10m), which provides 10-meter resolution global coverage of artificial surfaces with validated overall accuracy exceeding 86%[S40]; Copernicus DEM (topography), a 30-m resolution digital surface model (DSM) developed by the European Space Agency (ESA) through advanced radar satellite interferometry, widely recognized for its precision in geomorphological and environmental applications[S41]; Transportation intensity was characterized using a PCA-based composite index derived from OpenStreetMap (OSM) data; Industrial density was derived from a building function classification dataset using multi-source geospatial data[S9]; Population density was derived from a 100-m resolution gridded population dataset integrating China’s Seventh Census and Big Geospatial Data[S42].

All covariates were computed on a 1 km × 1 km grid. OSM point-of-interest (POI) densities were used to characterize traffic activity: for each grid cell, we counted POIs by category (covering parking/fuel and traffic-control/transport-stop features) and converted counts to densities by dividing by grid area; we then applied PCA to the category-density matrix and retained the first two components as traffic_density_PC1–PC2 to summarize multi-type traffic intensity while reducing collinearity. Road-network intensity was derived from OSM road polylines: for each road class, we summed total road length within each grid and normalized by grid area to obtain length densities. Rail-network intensity was computed analogously from OSM rail polylines, producing two area-normalized length-density variables for (i) conventional/intercity rail and (ii) urban rail transit (e.g., light rail/tram/monorail). PCA on the five class-wise length densities yielded road_network_PC1–PC3. Industrial intensity was measured as the area fraction of industrial-related building/facility polygons within each grid (industrial footprint area divided by grid area). Population density was obtained by aggregating a 100 m gridded population dataset to 1 km cells (sum of population within each 1 km grid) and dividing by grid area. Due to missing meteorological data, 2,083 UCS and 6,033 Non-UCS grids were included in the final matching (see Supplementary Table 20 in the separate file for more details). Matching was conducted nationally using the MatchIt package in R, with a caliper of 0.15 to ensure Adjusted standardized mean differences (Adjusted SMDs) below 0.1. Full covariate definitions, caliper settings, and balance statistics are reported in Supplementary Table 12.

To evaluate the effect of dust-proof net (DPN) coverage, we applied the similar matching procedure. UCS grids with DPN were labeled **DPN-UCS**, and those without as **Non-DPN-UCS**. The density of construction site area in 2020 was added as an additional covariate. We focused on 30 cities with >10% DPN coverage, yielding 612 DPN-UCS and 259 Non-DPN-UCS grids (see Supplementary Table 21 in the separate file for more details). Matching was again performed at the national level, with a caliper of 0.1 to ensure balance between groups.

Despite their limited surface area, construction activities significantly increased annual mean PM₂.₅ concentrations by 2.4% (*p* < 0.01) and PM₁₀ concentrations by 2.7% (*p* < 0.01) at the 1-km scale (Supplementary Fig. 14a). While the annual mean concentrations of PM₂.₅ and PM₁₀ were marginally lower in DPN-UCS grids than in Non-DPN-UCS grids (Supplementary Fig. 14b), the differences were not statistically significant after matching (PM₂.₅: *p* = 0.119; PM₁₀: *p* = 0.056), indicating that dust-proof net coverage did not lead to a meaningful reduction in ambient PM levels.

[Supplementary Table 12]

[Supplementary Fig. 14]

### **SR.2 Urban Construction Site Surrounding Nighttime Lighting Pollution**

Overlaying UCS distribution with high-resolution nighttime light imagery reveals a strong coupling between UCS and nighttime light radiance (Supplementary Fig. 15). It suggests that areas with higher nighttime light radiance around UCSs may impact the surrounding population.

[Supplementary Fig. 15]

### **SR.3 City-level UCS indicators**

To characterize city-level UCS activity and support the K-means typology, we define a compact set of summary indicators capturing counts, size structure, and area-normalized densities, together with basic demographics (Supplementary Table 13).

[Supplementary Table 13]

### **SR.4 Inspection of four types of urban renewal patterns**

As an external proxy, we use city yearbook "housing space under construction" (place-of-occurrence) [S43,44], which measures the total floor area actively under construction during the year and is conceptually consistent with our UCS site-based intensity indicators. Housing construction density was derived by normalizing these values by the urban area. Both the raw construction area and the derived construction density are used as external intensity proxies for the four types. To mitigate long-tailed distributions and outliers, both variables were log1p-transformed. A one-dimensional intensity index was then extracted from density and area via principle component analysis (PCA) and evaluated across the four groups using the Kruskal-Wallis test[S26]. Kruskal-Wallis tests indicate significant overall differences among the four typologies (Supplementary Table 14). Pairwise Mann–Whitney tests[S45], with Holm adjustment[S46] for multiple comparisons, are significant for every typology pairing (Supplementary Table 15).

[Supplementary Table 14]

[Supplementary Table 15]

## Supplementary Discussion

### **SD.1 Sensitivity analysis of morphological parameters**

High-resolution remote sensing image semantic segmentation operates at the pixel level, aiming to assign a land cover type to each pixel in the image[S47]. However, the resulting segmentation may contain isolated or incomplete regions. To enhance the integrity and accuracy of UCS parcels and ensure the final result reflects the true shape of the ground objects, we applied a series of morphological post-processing operations, including noise reduction, region merging, and hole filling. During region merging, exposed areas and DNP-covered areas within a specified buffer zone are assigned to a single UCS parcel.

According to the 2016 revised edition of the Urban Road Engineering Design Specifications (CJJ 37-2012), urban roads are classified into freeways, arterials, collectors, and local roads[S48]. According to the Urban Road Spatial Planning and Design Specifications of Beijing, generally, the right-of-way width of local roads is less than 25 meters, while the right-of-way widths of freeways, arterials and collectors are greater than 25 meters[S49]. A buffer-radius sensitivity analysis for parcel counts by size (Supplementary Table 9) and pixel-level accuracy on the 500-tile nationwide test set (Supplementary Table 10) support that 25 m as a stable default that balances removing spurious micro-fragments and avoiding over-merging. On this basis, we conducted multi-scale visual analysis. We visualized the UCS under different buffer zone parameters in Beijing and Shanghai. As shown in Supplementary Fig. 16, when the buffer zone distance is 10 meters, small fragmented patches are not effectively merged, leading to patches that should belong to the same UCS being split into multiple UCS. When the buffer zone distance is between 50 and 200 meters, some patches that should not belong to the same UCS are overly merged.

Given that roads in residential areas are primarily local roads, and based on manual visual interpretation results, in this study, we chose 25 meters as the buffer zone parameter for UCS merging. This buffer zone can effectively merge adjacent construction areas while avoiding excessive merging that may lead to information loss, ensuring the accuracy and rationality of the data.

[Supplementary Fig. 16]

[Supplementary Fig. 17-26]

**REFERENCE**

S1. Cao Y, Huang X. A coarse-to-fine weakly supervised learning method for green plastic cover segmentation using high-resolution remote sensing images. *ISPRS J Photogramm Remote Sens* 2022; **188**: 157–76.

S2. Huang X, Wang W, Li J *et al.* A Stepwise Refining Image-Level Weakly Supervised Semantic Segmentation Method for Detecting Exposed Surface for Buildings (ESB) From Very High-Resolution Remote Sensing Images. *IEEE Trans Geosci Remote Sensing* 2024; **62**: 1–17.

S3. Li X, Gong P, Zhou Y *et al.* Mapping global urban boundaries from the global artificial impervious area (GAIA) data. *Environ Res Lett* 2020; **15**: 94044.

S4. Li J, Liu T, Yang J *et al.* Mapping of 30m global urban boundaries from 1972 to 2021 based on multi-source geographic information fusion. [Data set]. *Zenodo*. https://doi.org/10.5281/zenodo.14523232.

S5. Yue K, Yang L, Li R *et al.* TreeUNet: Adaptive Tree convolutional neural networks for subdecimeter aerial image segmentation. *ISPRS J Photogramm Remote Sens* 2019; **156**: 1–13.

S6. Kirillov A, Mintun E, Ravi N *et al.* Segment Anything. In: *IEEE International Conference on Computer Vision*. Paris, France, 2023.

S7. Radosavovic I, Kosaraju RP, Girshick R *et al.* Designing network design spaces. In: *IEEE Computer Society Conference on Computer Vision and Pattern Recognition*. Seattle, USA, 2020.

S8. Yang L, Qi L, Feng L *et al.* Revisiting Weak-to-Strong Consistency in Semi-Supervised Semantic Segmentation. In: *IEEE Computer Society Conference on Computer Vision and Pattern Recognition*. Vancouver, Canada, 2023.

S9. Huang X, Zhang Z, Li J. China’s first sub-meter building footprints derived by deep learning. *Remote Sens Environ* 2024; **311**: 114274.

S10. Zhang Z, Qian Z, Zhong T *et al.* Vectorized rooftop area data for 90 cities in China. *Sci Data* 2022; **9**: 3–5.

S11. Liu Z, Tang H, Feng L *et al.* China Building Rooftop Area: the first multi-annual (2016-2021) and high-resolution (2.5m) building rooftop area dataset in China derived with super-resolution segmentation from Sentinel-2 imagery. *Earth Syst Sci Data* 2023; **15**: 3547–72.

S12. Zhou W, Yu W, Qian Y *et al.* Beyond city expansion: multi-scale environmental impacts of urban megaregion formation in China. *Natl Sci Rev* 2021; **9**: nwab107.

S13. Law insider. *URBAN CONSTRUCTION SITE Definition*. https://www.lawinsider.com/dictionary/urban-construction-site (6 February 2026, date last accessed).

S14. Hare WL, Koch VR, Lucet Y. Models and algorithms to improve earthwork operations in road design using mixed integer linear programming. *Eur J Oper Res* 2011; **215**: 470–80.

S15. Luo Q, Huang L, Xue X *et al.* Occupational health risk assessment based on dust exposure during earthwork construction. *J Build Eng* 2021; **44**: 103186.

S16. Liu C, Feng Q, Liu J *et al.* Urban green plastic cover extraction and spatial pattern changes in Jinan city based on DeepLabv3+ semantic segmentation model. *Nat Remote Sens Bull* 2022; **26**: 2518–30.

S17. Liu J, Feng Q, Wang Y *et al.* Urban green plastic cover mapping based on VHR remote sensing images and a deep semi-supervised learning framework. *ISPRS Int J Geo-Inf* 2020; **9**: 527.

S18. Sun X, Yin D, Qin F *et al.* Revealing influencing factors on global waste distribution via deep-learning based dumpsite detection from satellite imagery. *Nat Commun* 2023; **14**: 1–13.

S19. Xie E, Wang W, Yu Z *et al.* SegFormer: Simple and Efficient Design for Semantic Segmentation with Transformers. In: *Advances in Neural Information Processing Systems*. Virtual, 2021.

S20. Minaee S, Boykov Y, Porikli F *et al.* Image Segmentation Using Deep Learning: A Survey. *IEEE Trans Pattern Anal Mach Intell* 2022; **44**: 3523–42.

S21. Velastegui R, Tatarchenko M, Karaoglu S *et al.* Image semantic segmentation of indoor scenes: A survey. *Comput Vis Image Underst* 2024; **248**: 104102.

S22. Pandey S, Chen KF, Dam EB. Comprehensive Multimodal Segmentation in Medical Imaging: Combining YOLOv8 with SAM and HQ-SAM Models. In: *IEEE/CVF International Conference on Computer Vision Workshops*. Paris, France, 2023.

S23. Wang J, Sun K, Cheng T *et al.* Deep High-Resolution Representation Learning for Visual Recognition. *IEEE Trans Pattern Anal Mach Intell* 2021; **43**: 3349–64.

S24. Zhang H, Hu W, Wang X. ParC-Net: Position Aware Circular Convolution with Merits from ConvNets and Transformer. In: *European Conference on Computer Vision*. Tel Aviv, Israel, 2022.

S25. Zhang W, Huang Z, Luo G *et al.* TopFormer: Token Pyramid Transformer for Mobile Semantic Segmentation. In: *IEEE Computer Society Conference on Computer Vision and Pattern Recognition*. New Orleans, USA, 2022.

S26. Kruskal WH, Wallis WA. Use of ranks in one-criterion variance analysis. *J Am Stat Assoc* 1952; **47**: 583–621.

S27. Standing Committee of the Shenzhen Municipal People’s Congress. *Shenzhen Special Economic Zone Urban Renewal Regulations*. https://www.sz.gov.cn/szsrmzfxxgk/zc/gz/content/post_9453864.html (6 February 2026, date last accessed).

S28. Ministry of Housing and Urban–Rural Development. *Notice of the Ministry of Housing and Urban–Rural Development on Preventing Large-Scale Demolition and Construction during Urban Renewal Actions*. https://www.mohurd.gov.cn/gongkai/zc/wjk/art/2021/art_17339_761887.html (6 February 2026, date last accessed).

S29. Shenzhen Municipal Housing and Construction Bureau. *Notice of the General Office of the Shenzhen Municipal People’s Government on Printing and Distributing the “Implementation Opinions on Actively and Steadily Promoting Urban Village Redevelopment to Achieve High-Quality Development.”* https://www.sz.gov.cn/cn/xxgk/zfxxgj/zcfg/content/post_11274442.html (6 February 2026, date last accessed).

S30. Shenzhen Municipal Housing and Construction Bureau. *Notice of the Shenzhen Municipal Housing and Construction Bureau on Issuing the “Several Opinions on Regulating the Implementation of Urban Renewal.”* https://www.sz.gov.cn/cn/xxgk/zfxxgj/zcfg/content/post_12090584.html (6 February 2026, date last accessed).

S31. Jiang N, Dong Z, Xu Y *et al.* Characterization of PM10and PM2.5source profiles of fugitive dust in Zhengzhou, China. *Aerosol Air Qual Res* 2018; **18**: 314–29.

S32. Du Y, Ren H, Cai W *et al.* Effect of construction dust on urban PM2.5 emission characteristics: A case study of the main urban area of Chongqing, China. *Nat Environ Pollut Technol* 2016; **15**: 833–40.

S33. Kinsey JS, Cowherd C. Particulate emissions from construction activities. *J Air Waste Manage Assoc* 2005; **55**: 772–83.

S34. Zhang C, Chen Z, Luo L *et al.* Mapping urban construction sites in China through geospatial data fusion: Methods and applications. *Remote Sens Environ* 2024; **315**: 114441.

S35. Yang J, Huang X. The 30m annual land cover dataset and its dynamics in China from 1990 to 2019. *Earth Syst Sci Data* 2021; **13**: 3907–25.

S36. Davis KF, Yu K, Rulli MC *et al.* Accelerated deforestation driven by large-scale land acquisitions in Cambodia. *Nat Geosci* 2015; **8**: 772–5.

S37. Wei J, Li Z, Lyapustin A *et al.* Reconstructing 1-km-resolution high-quality PM2.5 data records from 2000 to 2018 in China: spatiotemporal variations and policy implications. *Remote Sens Environ* 2021; **252**: 112136.

S38. Wei J, Li Z, Xue W *et al.* The ChinaHighPM10 dataset: generation, validation, and spatiotemporal variations from 2015 to 2019 across China. *Environ Int* 2021; **146**: 106290.

S39. Hu Y, Zhang L, Zhao Y *et al.* ChinaMet: A multisource integrated high-resolution multi-variable meteorological dataset for China. [Data set]. *Zenodo*. https://doi.org/10.5281/zenodo.14580706.

S40. Huang X, Yang J, Wang W *et al.* Mapping 10 m global impervious surface area (GISA-10m) using multi-source geospatial data. *Earth Syst Sci Data* 2022; **14**: 3649–72.

S41. Fahrland, E. *Copernicus Dem Product Handbook (v3. 0)*. https://object.cloud.sdsc.edu/v1/AUTH_opentopography/www/metadata/Copernicus_metadata.pdf (6 February 2026, date last accessed).

S42. Chen Y, Xu C, Ge Y *et al.* A 100 m gridded population dataset of China’s seventh census using ensemble learning and big geospatial data. *Earth Syst Sci Data* 2024; **16**: 3705–18.

S43. Beijing Municipal Bureau of Statistics. *Beijing Statistical Yearbook*. https://nj.tjj.beijing.gov.cn/nj/main/2021-tjnj/zk/e/indexch.htm (6 February 2026, date last accessed).

S44. Shanghai Municipal Bureau of Statistics. *Shanghai Statistical Yearbook*. https://tjj.sh.gov.cn/tjnj/20220309/0e01088a76754b448de6d608c42dad0f.html (6 February 2026, date last accessed).

S45. Mann HB, Whitney DR. On a test of whether one of two random variables is stochastically larger than the other. *Ann Math Stat* 1947: 50–60.

S46. Holm S. A simple sequentially rejective multiple test procedure. *Scand J Stat* 1979; **6**: 65–70.

S47. Li Y, Shi T, Zhang Y *et al.* Learning deep semantic segmentation network under multiple weakly-supervised constraints for cross-domain remote sensing image semantic segmentation. *ISPRS J Photogramm Remote Sens* 2021; **175**: 20–33.

S48. Ministry of Housing and Urban-Rural Development. *Urban Road Engineering Design Specifications (CJJ 37-2012)*. https://www.mohurd.gov.cn/gongkai/zc/wjk/art/2016/art_17339_228082.html (6 February 2026, date last accessed).

S49. Beijing Municipal Commission of Planning and Natural Resources. *Urban Road Spatial Planning and Design Specifications of Beijing*. https://ghzrzyw.beijing.gov.cn/biaozhunguanli/bz/szjgdjt/202406/P020240603566610400349.pdf (6 February 2026, date last accessed).

**Figure and Table Captions**


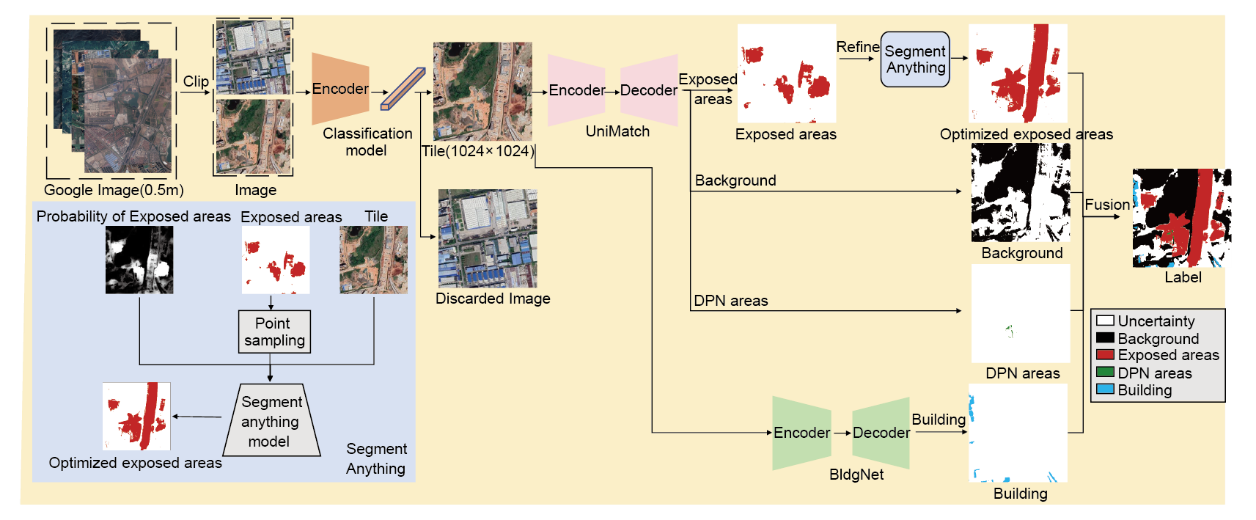


**Supplementary Fig. 1. The proposed semi-automatic annotation approach.** The blue box depicts the process of optimizing exposed areas detection using Segment Anything model (SAM). 'Probability' represents the classification scores from UniMatch for the exposed areas detection, where brighter colors (closer to white) indicate higher probabilities. Image data © Google.


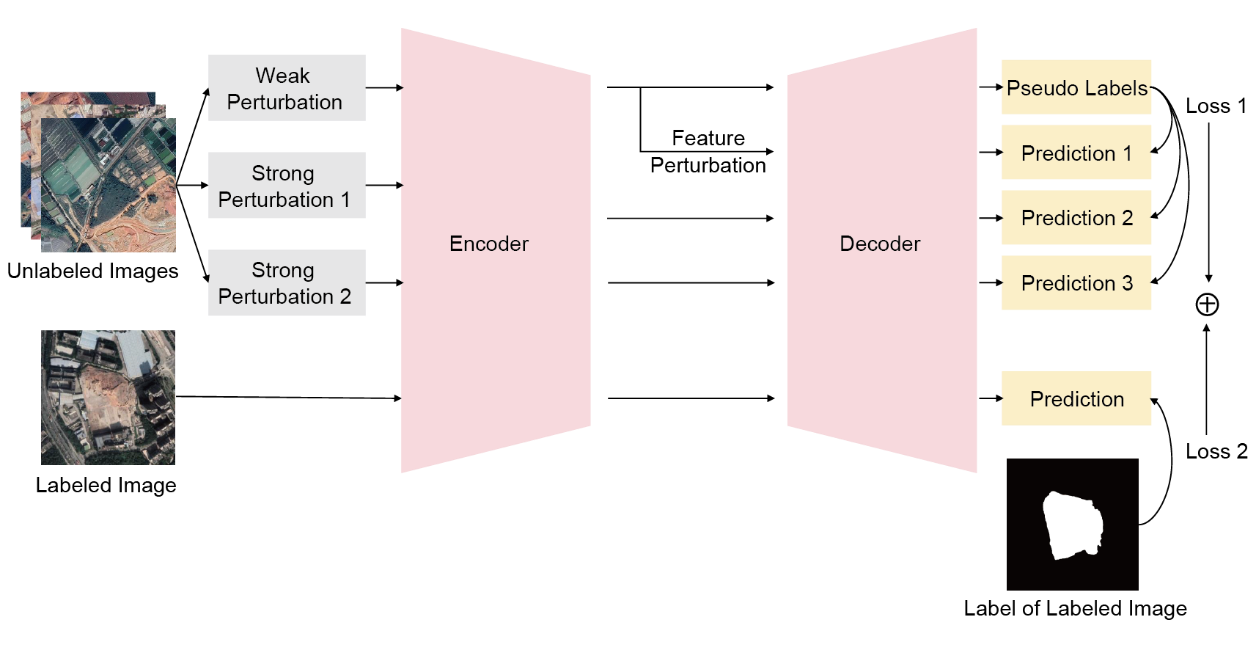


**Supplementary Fig. 2. The Flowchart of UniMatch.** Weak perturbation involves basic augmentation operations on unlabeled images, such as rotation and flipping. Strong perturbation applies more complex augmentations, including color jittering, blurring, and grayscaling. Feature perturbation introduces dropout (rate: 0.5) to the features generated by the encoder. Image data © Google.


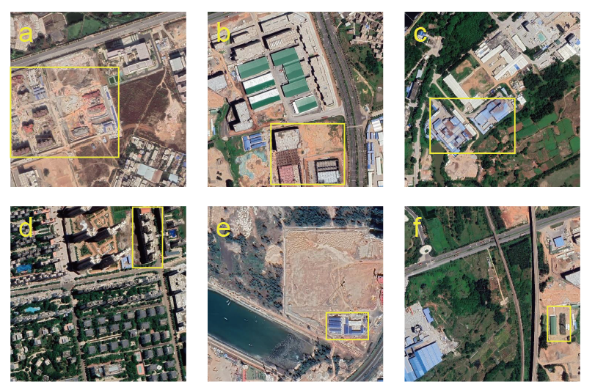


**Supplementary Fig.3: Buildings and UCSs. a-b** Buildings (highlighted in yellow boxes) under construction. **c-d** Buildings (highlighted in yellow boxes) spatially co-located with UCSs. **e-f** Temporary structures (highlighted in yellow boxes) located on UCSs. Image data © Google.


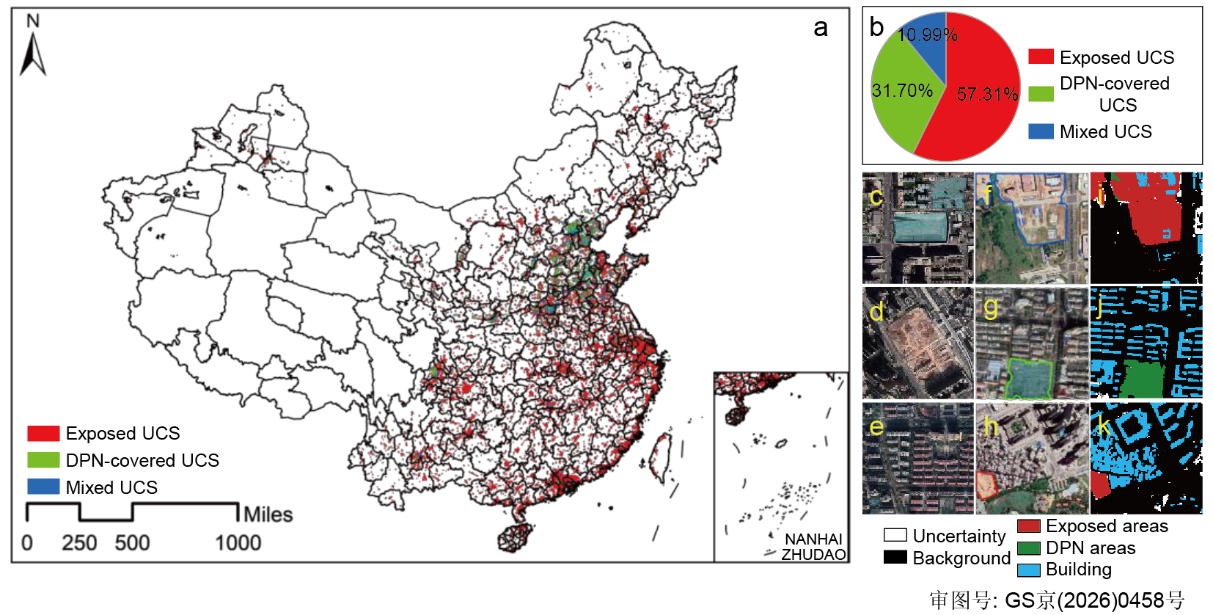


**Supplementary Fig.4: Overview of the sample dataset and representative UCS examples. a** Geographical distribution of the semi-automatic sample dataset, **b** Composition of the sample dataset, **c-e** Examples of image-level samples (DPN image, exposed image, and background image) sized 0.5 km × 0.5 km, **f-h** UCS satellite images highlighted with colored boundaries for the semi-automatically collected mixed UCS, DPN UCS, and exposed UCS, respectively, **i-k** Pixel-wise foreground annotations corresponding to the satellite images in **f-h.** Image data © Google.


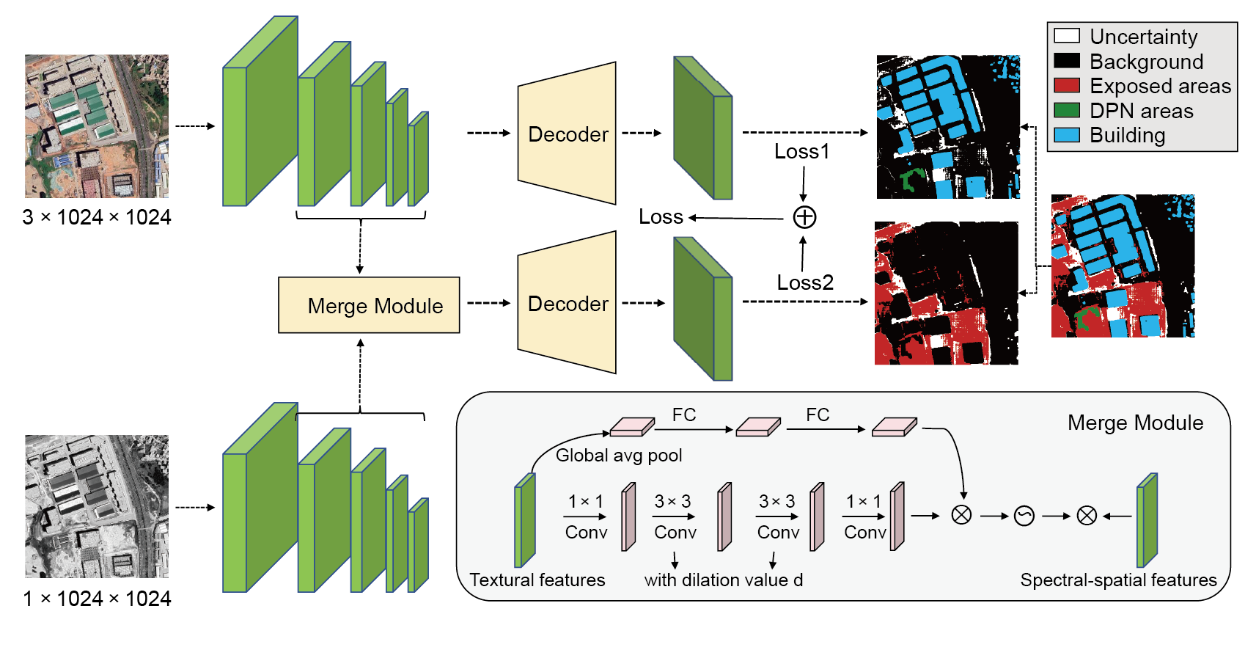


**Supplementary Fig. 5. The proposed two-branch sematic segmentation model.** Image data © Google.


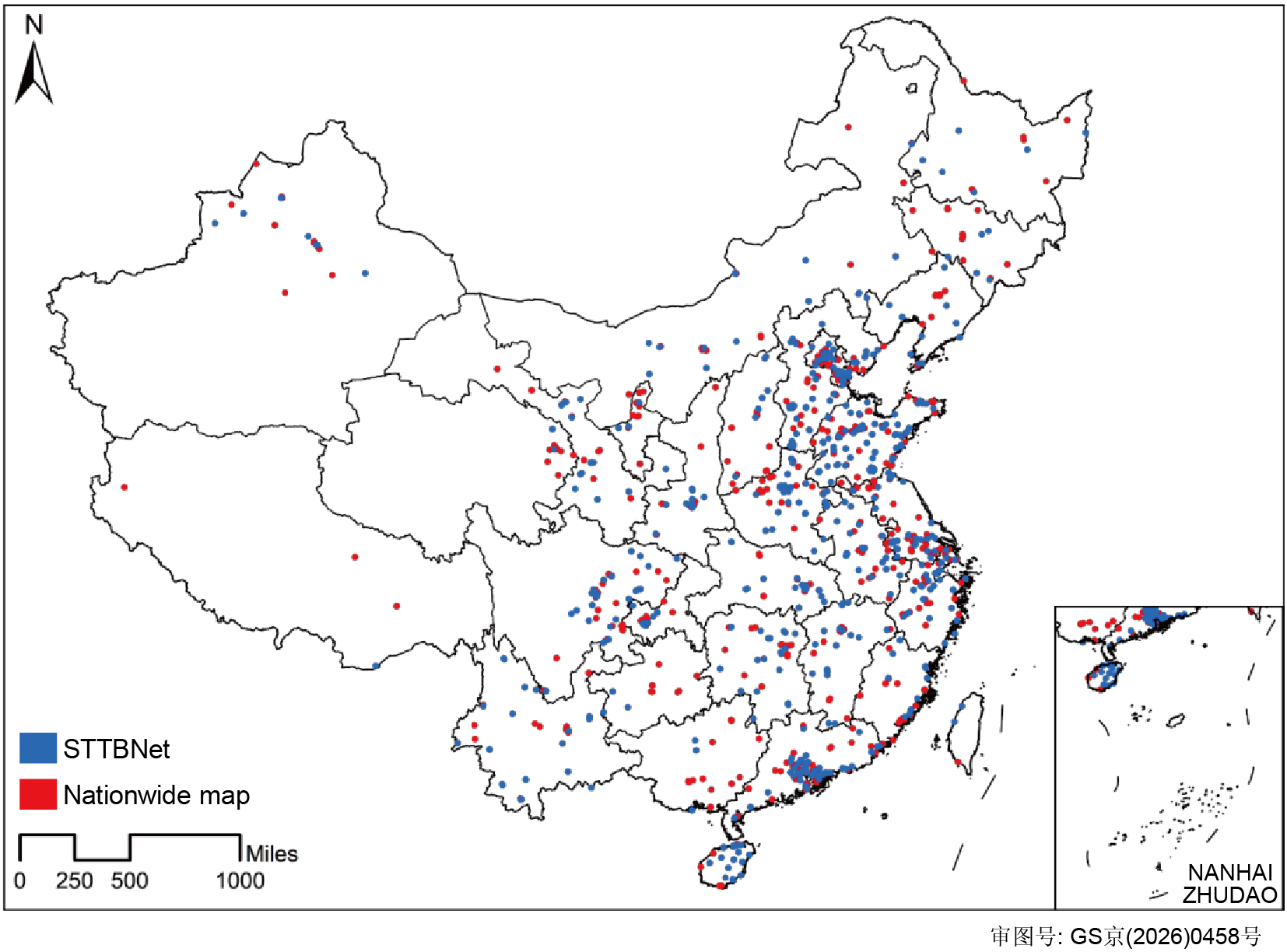


**Supplementary Fig. 6. The distribution of test samples for STTBNet (blue) and the nationwide map (red).**


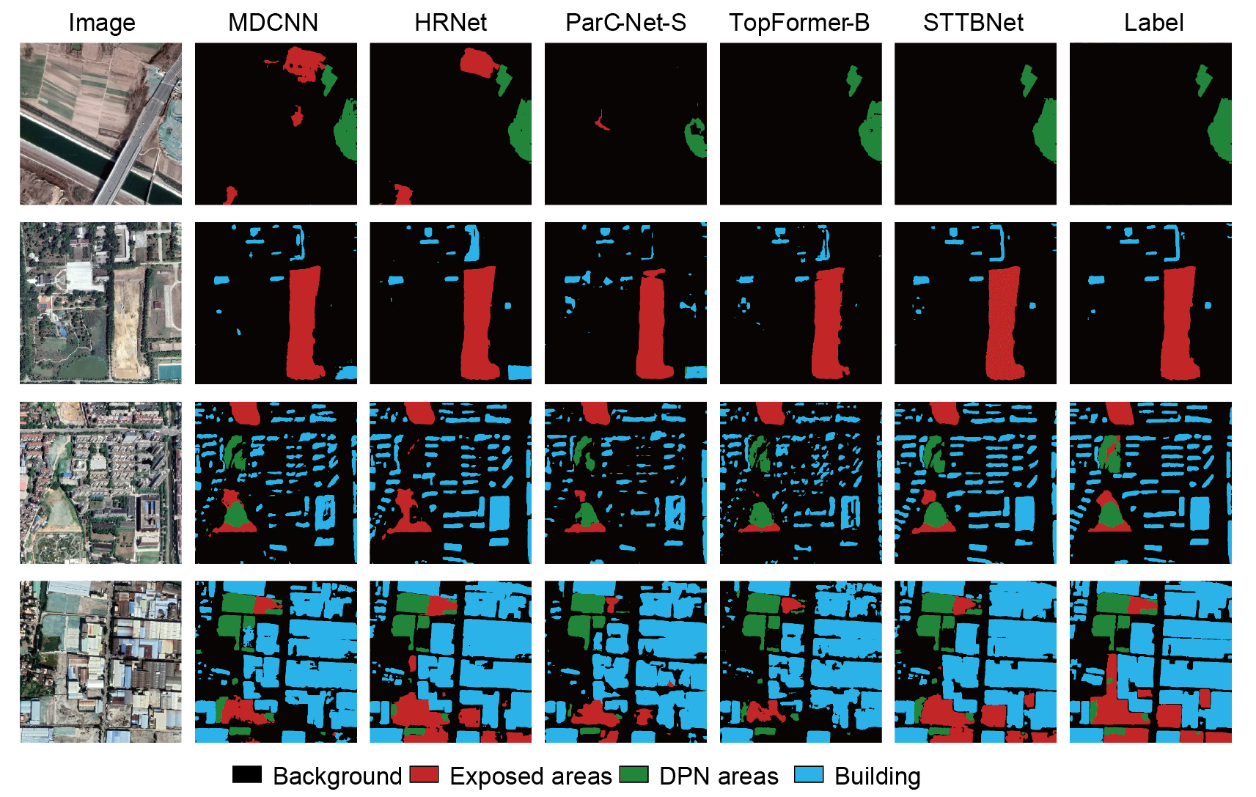


**Supplementary Fig. 7. Visualization results of different models on the test samples.** Image data © Google.


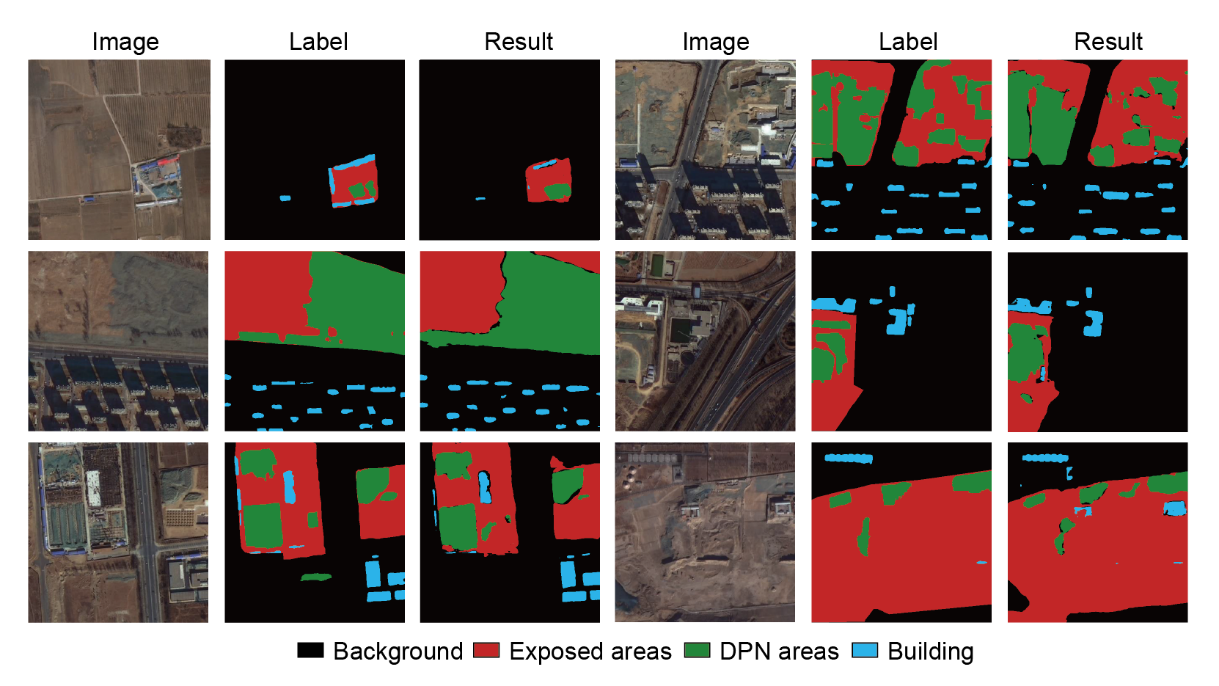


**Supplementary Fig. 8**. **Qualitative results of UCS segmentation on GF-2 imagery (Beijing, 2015)**: true color GF-2 image, Reference label, and STTBNet prediction.


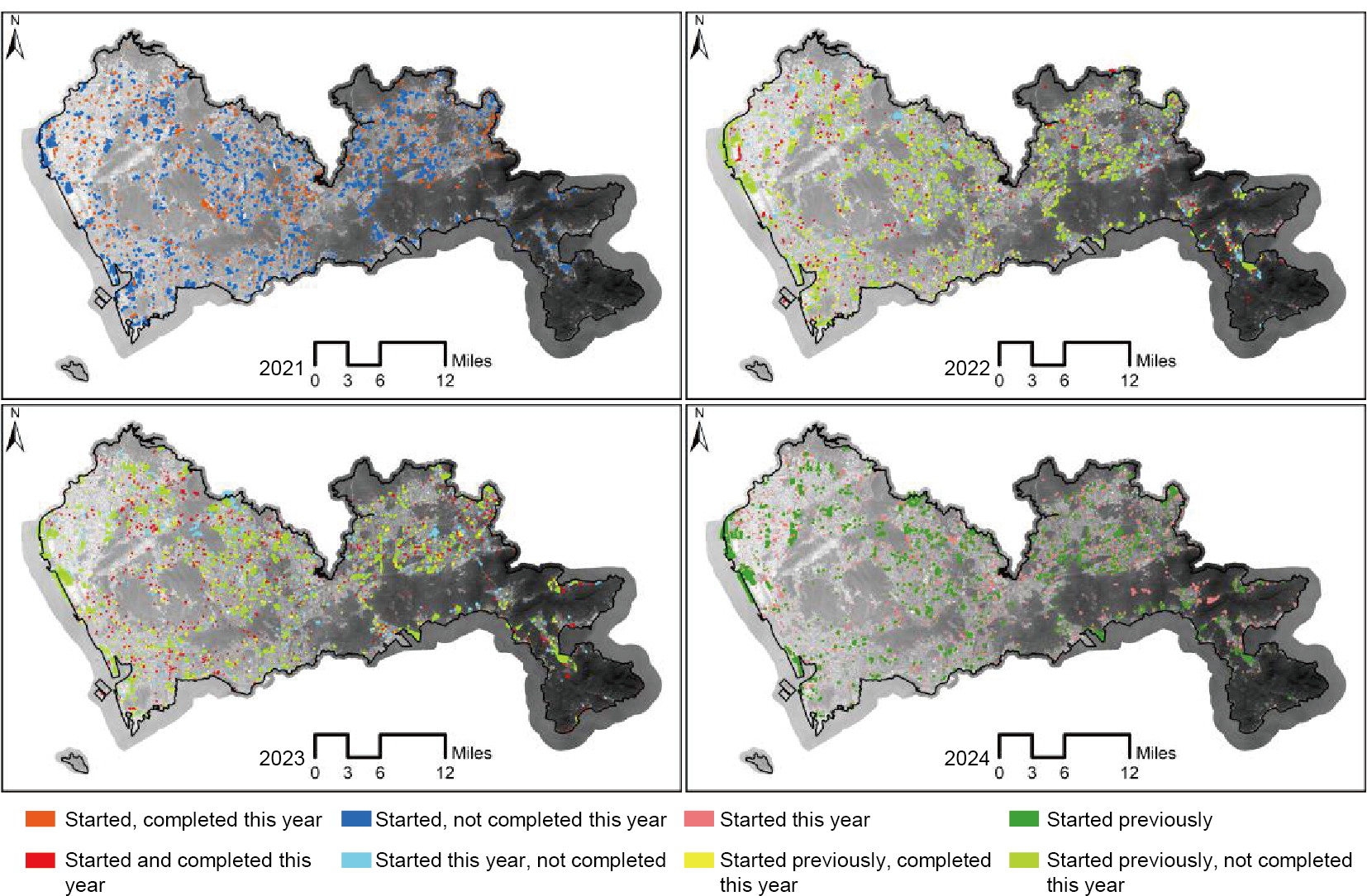


**Supplementary Fig. 9. Multi-year UCS mapping in Shenzhen (2021–2024) showing annual UCS status and inter-annual site trajectories derived by parcel linking**


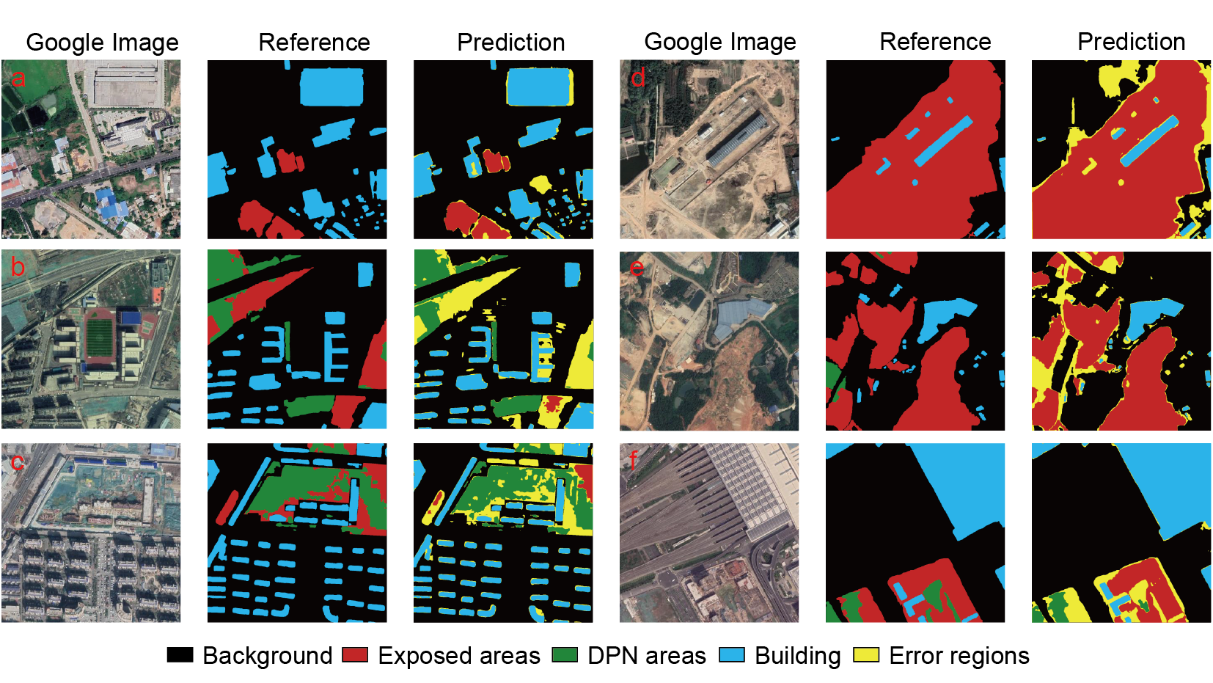


**Supplementary Fig. 10**. **Typical failure modes of UCS segmentation on Google Earth imagery**: true color image, Reference label, and STTBNet prediction. Image data © Google.


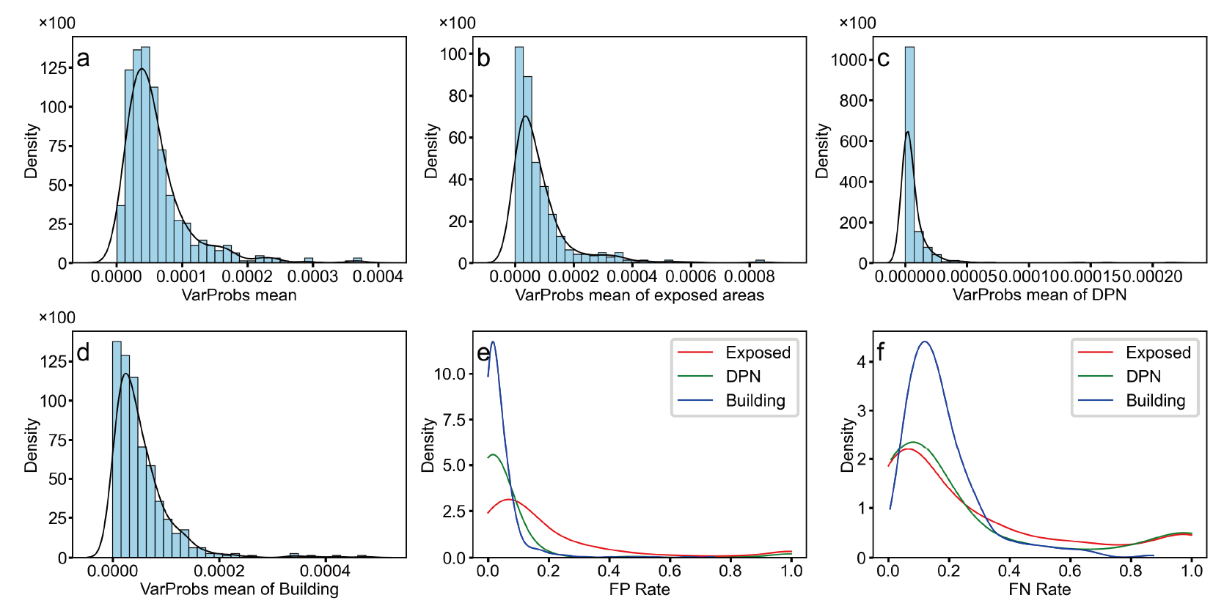


**Supplementary Fig. 11. Statistical distributions of predictive uncertainty and class-wise error rates on the test images.** Panels **a–d** present the image-level uncertainty metric VarProbs mean, computed respectively over all pixels, exposed areas, DPN-covered areas, and buildings. Panel **e** reports the class-wise false positive rate (FP rate), while panel **f** reports the class-wise false negative rate (FN rate). In all subplots, the y-axis represents probability density, and the “×100” label indicates that the density values on the vertical axis have been rescaled.


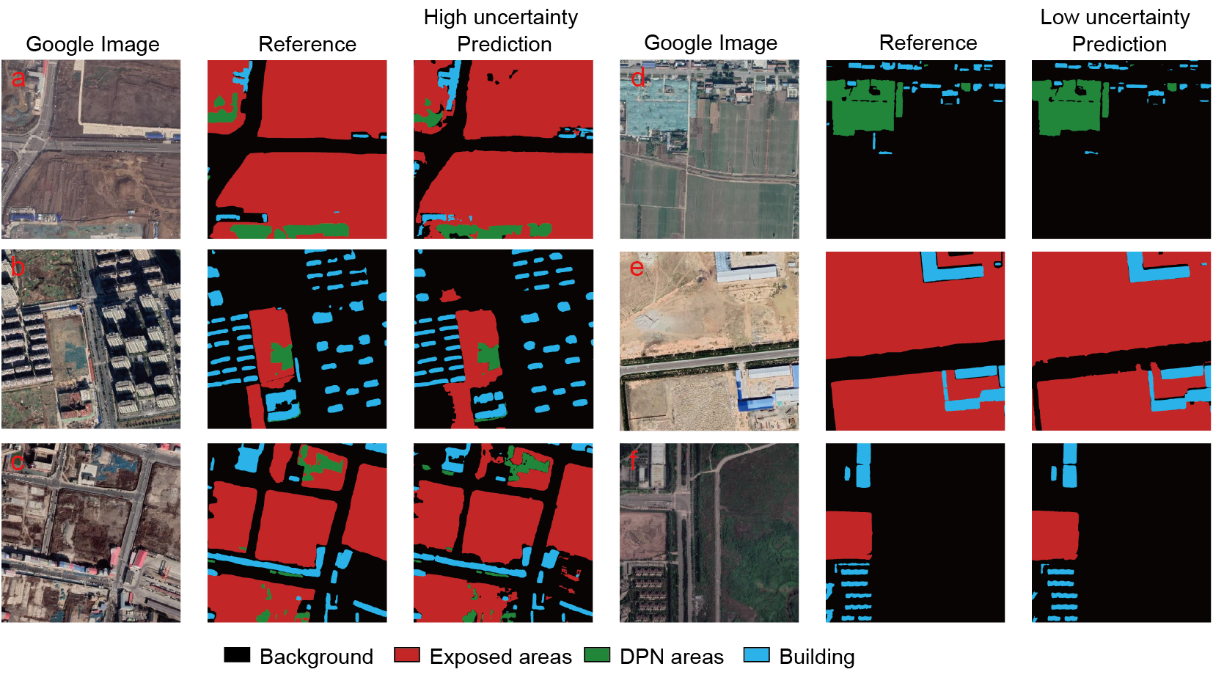


**Supplementary Fig. 12. Visual samples with low and high predictive uncertainty under Monte-Carlo dropout.** Image data © Google.


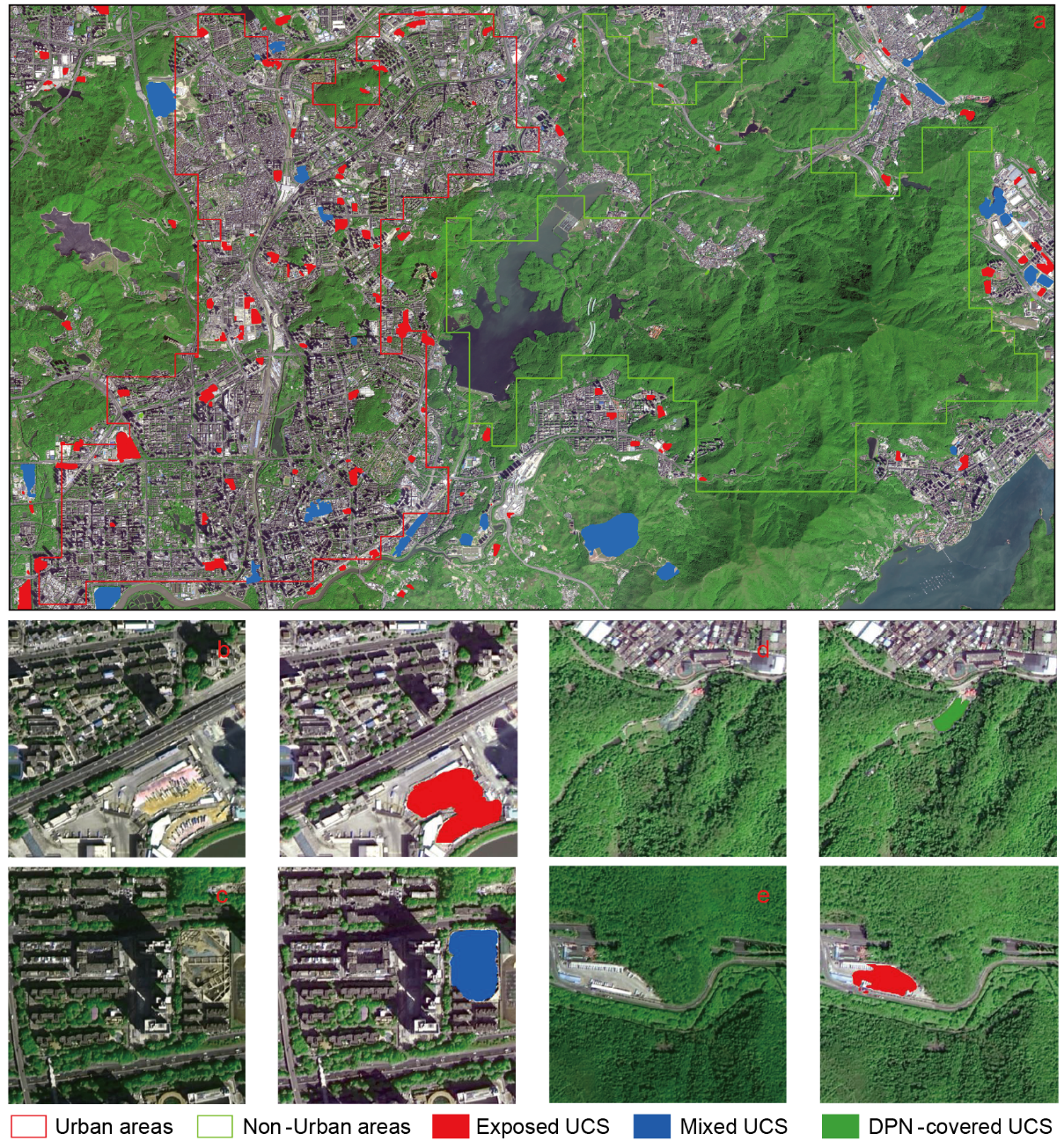


**Supplementary Fig. 13. Urban and non-urban negative-control check in Shenzhen (2022).** **a** maps UCS predictions in the Wutongshan scenic area (predominantly non-urban) and the adjacent urban region, with the non-urban mask indicated. **b–c** show representative urban tiles, including true detections (in c) and rare false alarms (in b) for visual reference. **d-e** shows representative non-urban tiles from Wutongshan, including true detections (in d) and rare false alarms (in e) for visual reference.


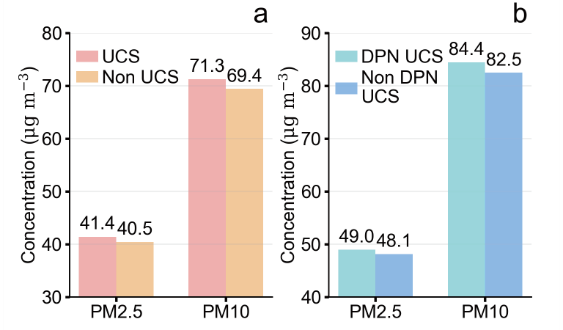


**Supplementary Fig. 14. The impact of UCS on the environment. a** Annual mean concentrations of PM₂.₅ and PM₁₀ for matched UCS grids and Non-UCS grids. **b** Annual mean concentrations of PM₂.₅ and PM₁₀ for matched DPN-UCS grids and Non-DPN-UCS grids.


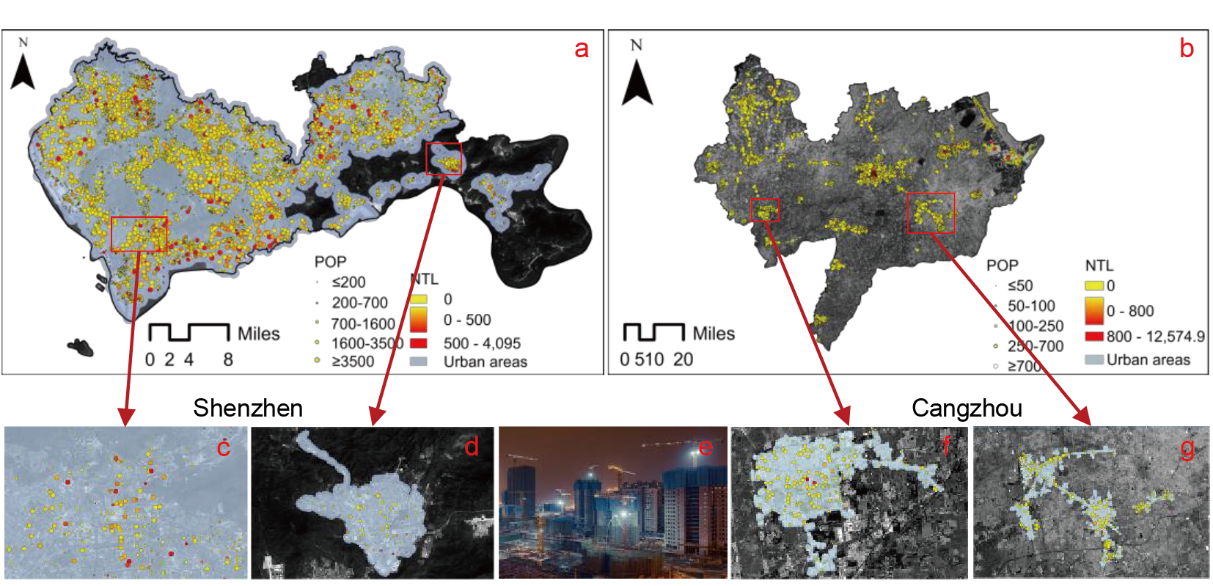


**Supplementary Fig. 15. Urban Construction Site Surrounding Nighttime Lighting and Population.** Current Studies indicate that strong NTL within 200–500m of residences can significantly impact sleep quality and melatonin secretion. Here POP refers to the total population within a 200-m radius around each UCS, NTL represents the nighttime radiance intensity with the unit of nW×cm^-2^×sr^-1^ at each UCS. **c** and **d** show local zoom-in regions from the megacity Shenzhen (**a**), while **f** and **g** depict zoom-in regions from the medium-sized city Cangzhou. **e** shows an aerial view of NTL at the UCS.


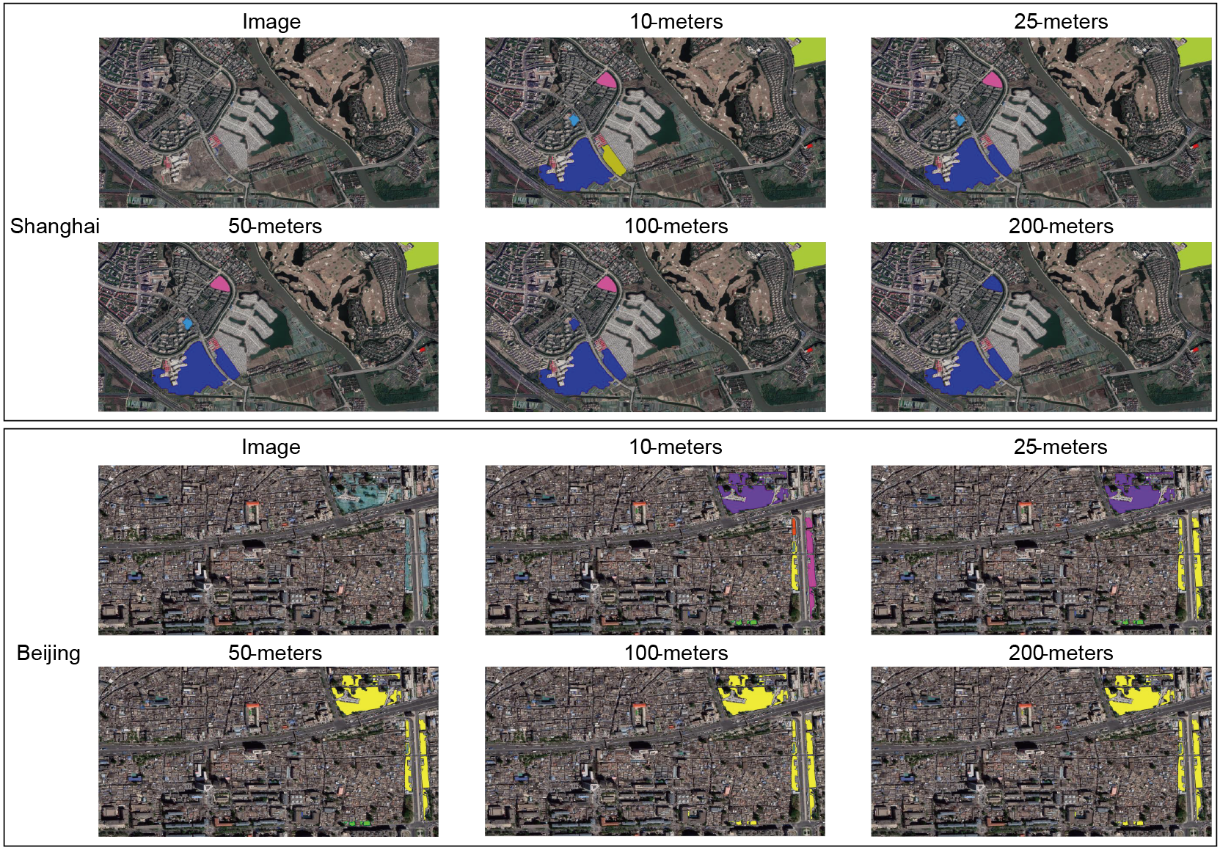


**Supplementary Fig. 16. UCS visualization under different buffer zone parameters.** Each color represents a different UCS. Image data © Google.


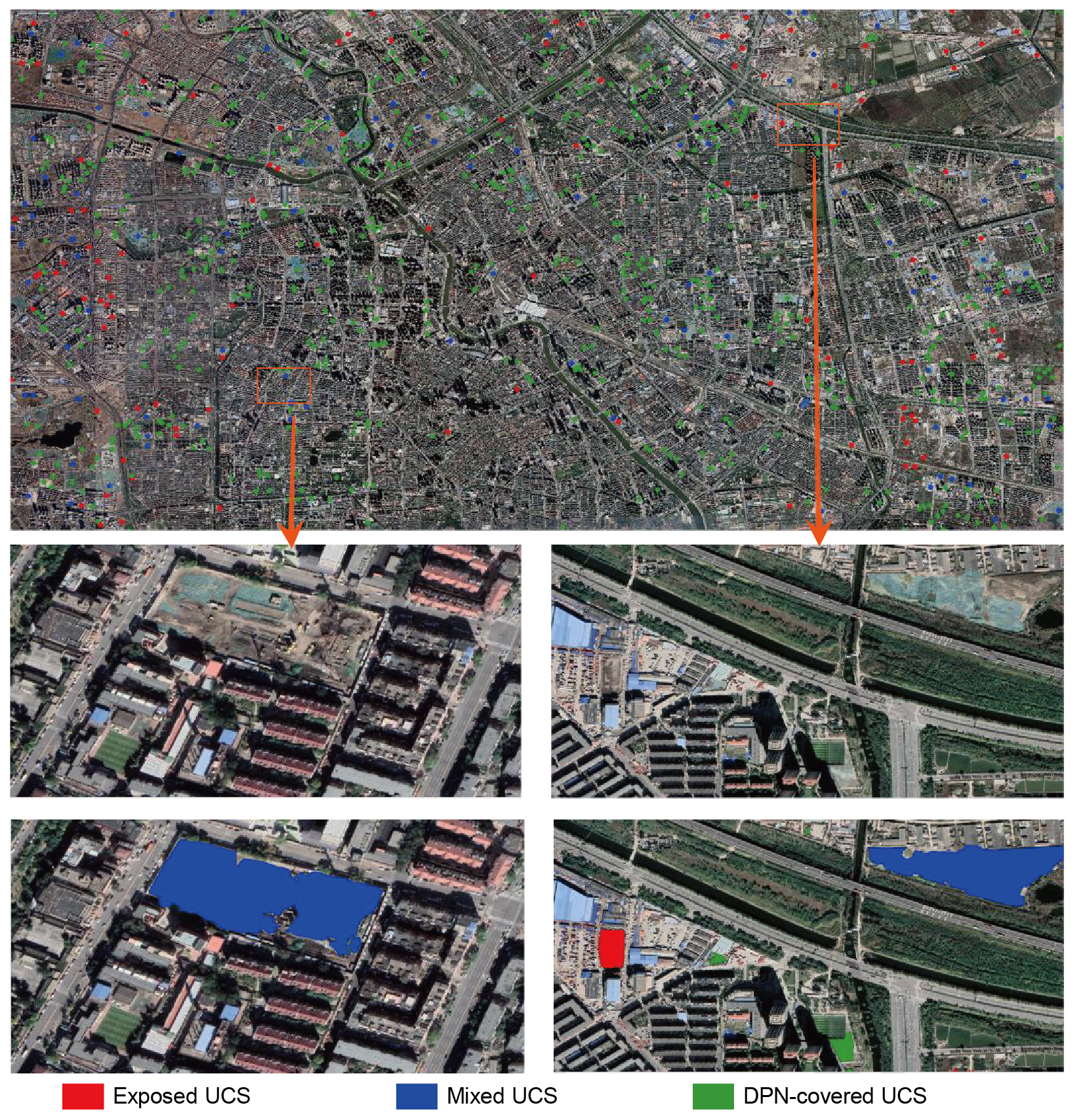


**Supplementary Fig. 17. UCS distribution in the core of Tianjin.** Tianjin is one of the mega cities. Image data © Google.


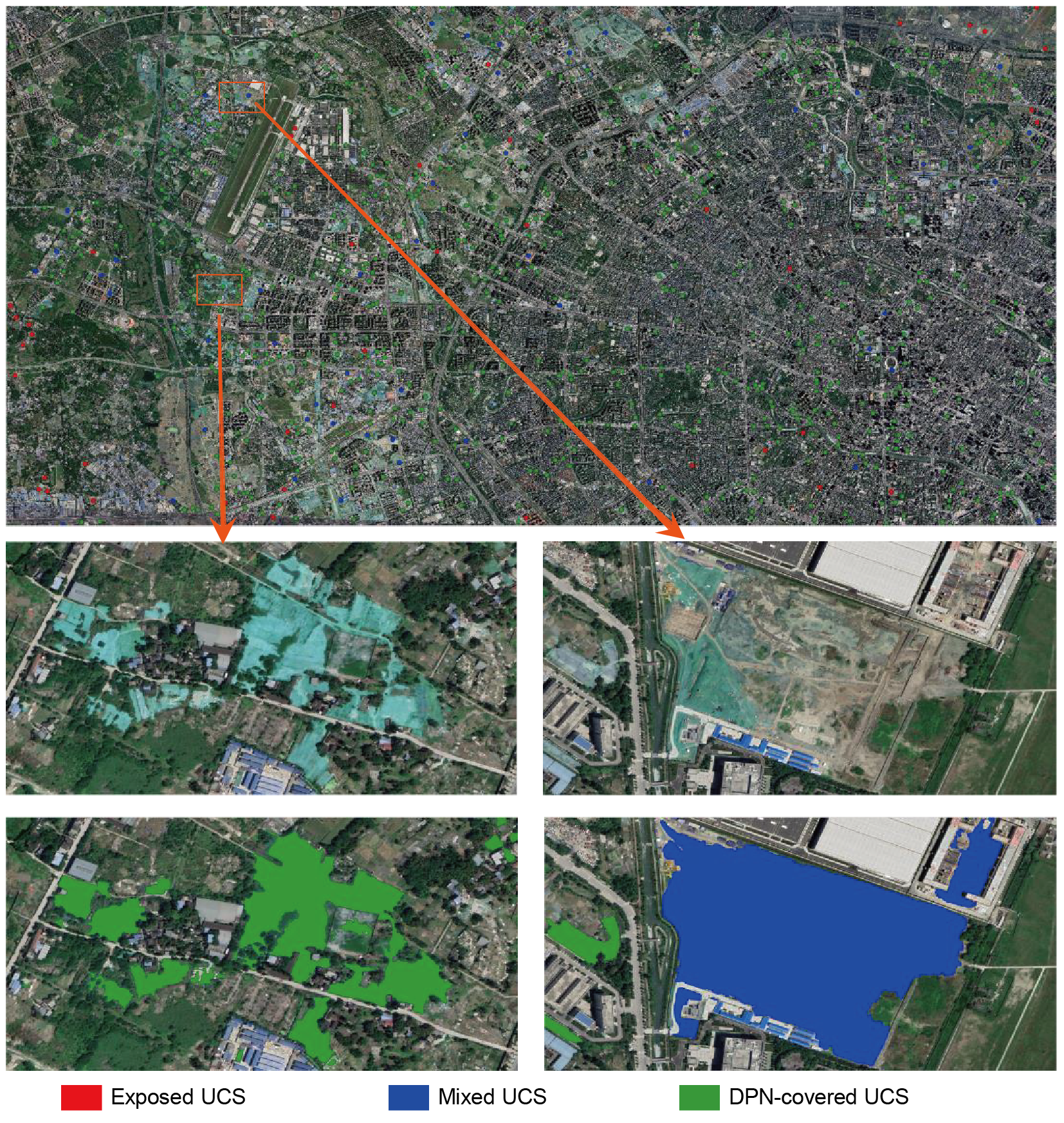


**Supplementary Fig. 18. UCS distribution in the fringe of Chengdu.** Chengdu is one of the mega cities. Image data © Google.


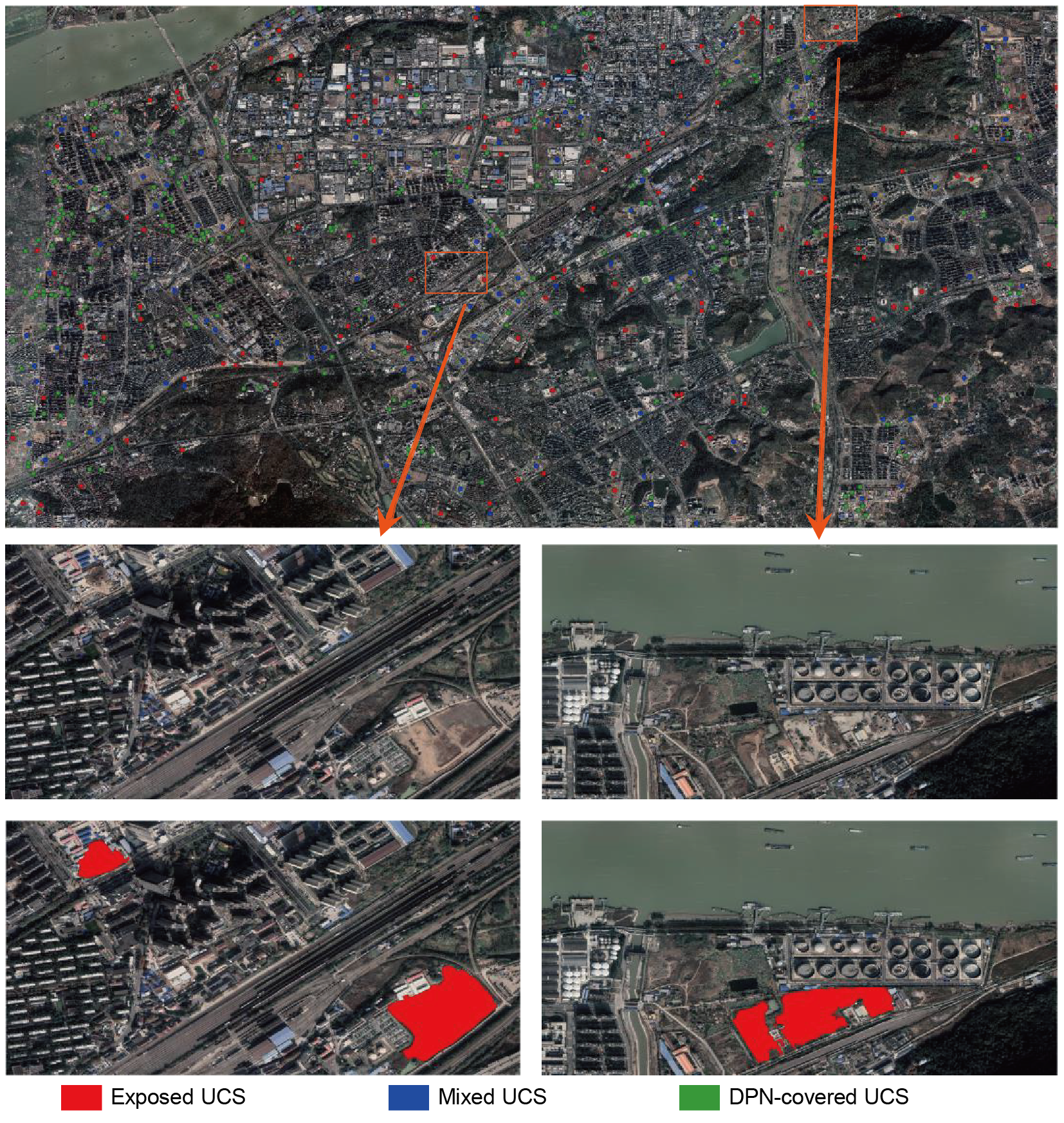


**Supplementary Fig. 19. UCS distribution in the fringe of Nanjing.** Nanjing is one of the super cities. Image data © Google.


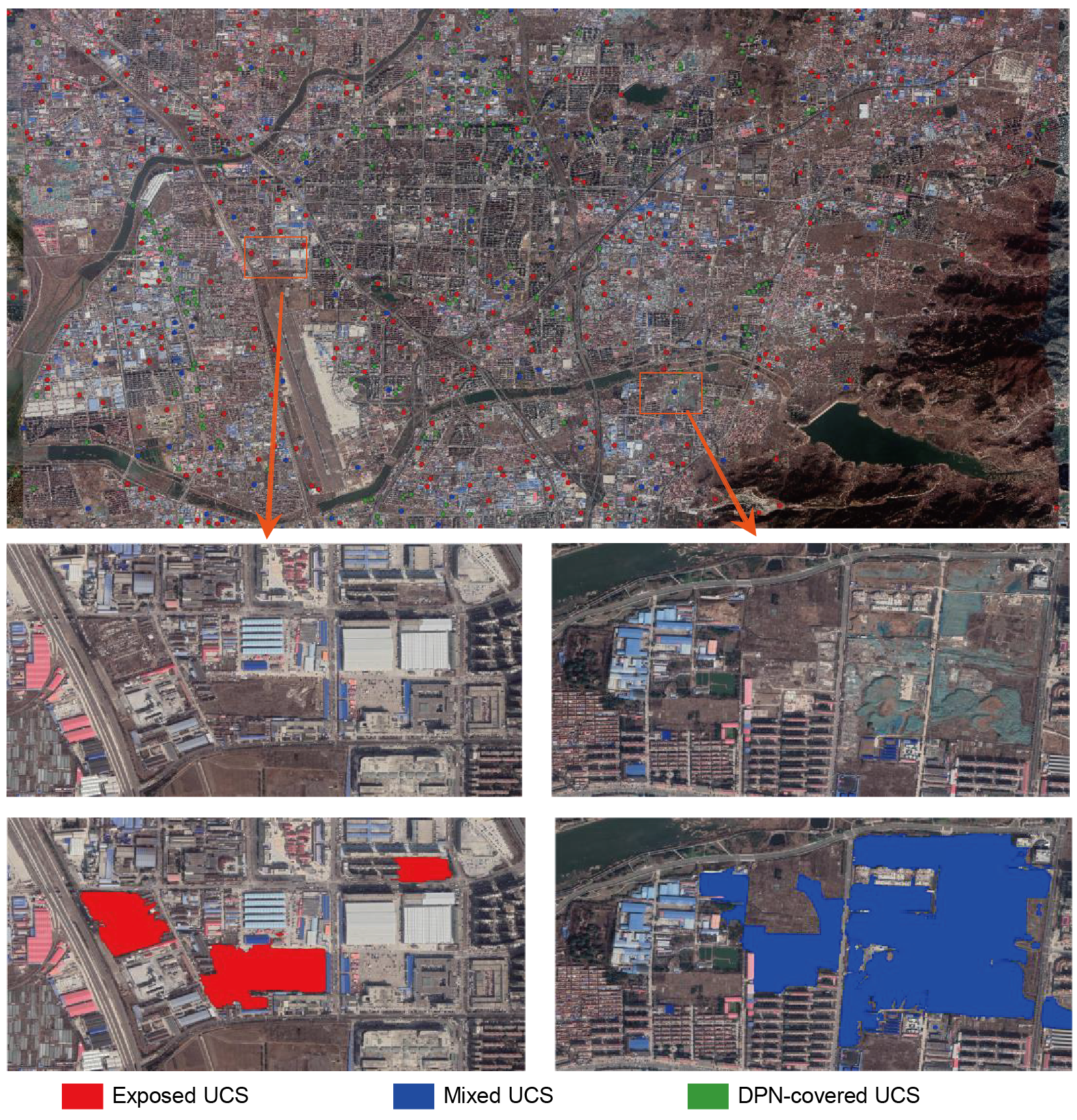


**Supplementary Fig. 20. UCS distribution in the core of Qingdao.** Qingdao is one of the super cities. Image data © Google.


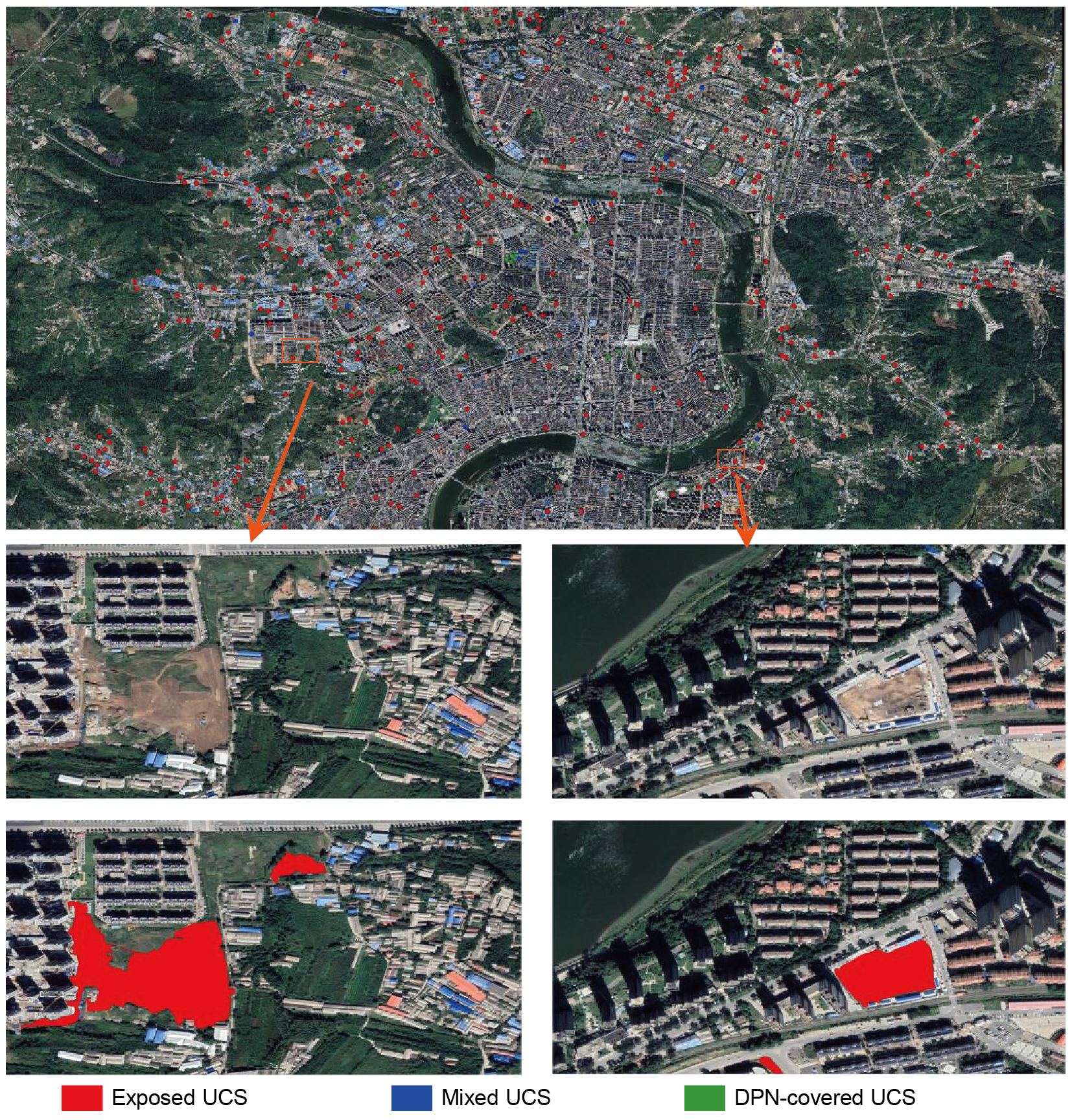


**Supplementary Fig. 21. UCS distribution in the new town of** **Jilin**. Jilin is one of the big cities. Image data © Google.


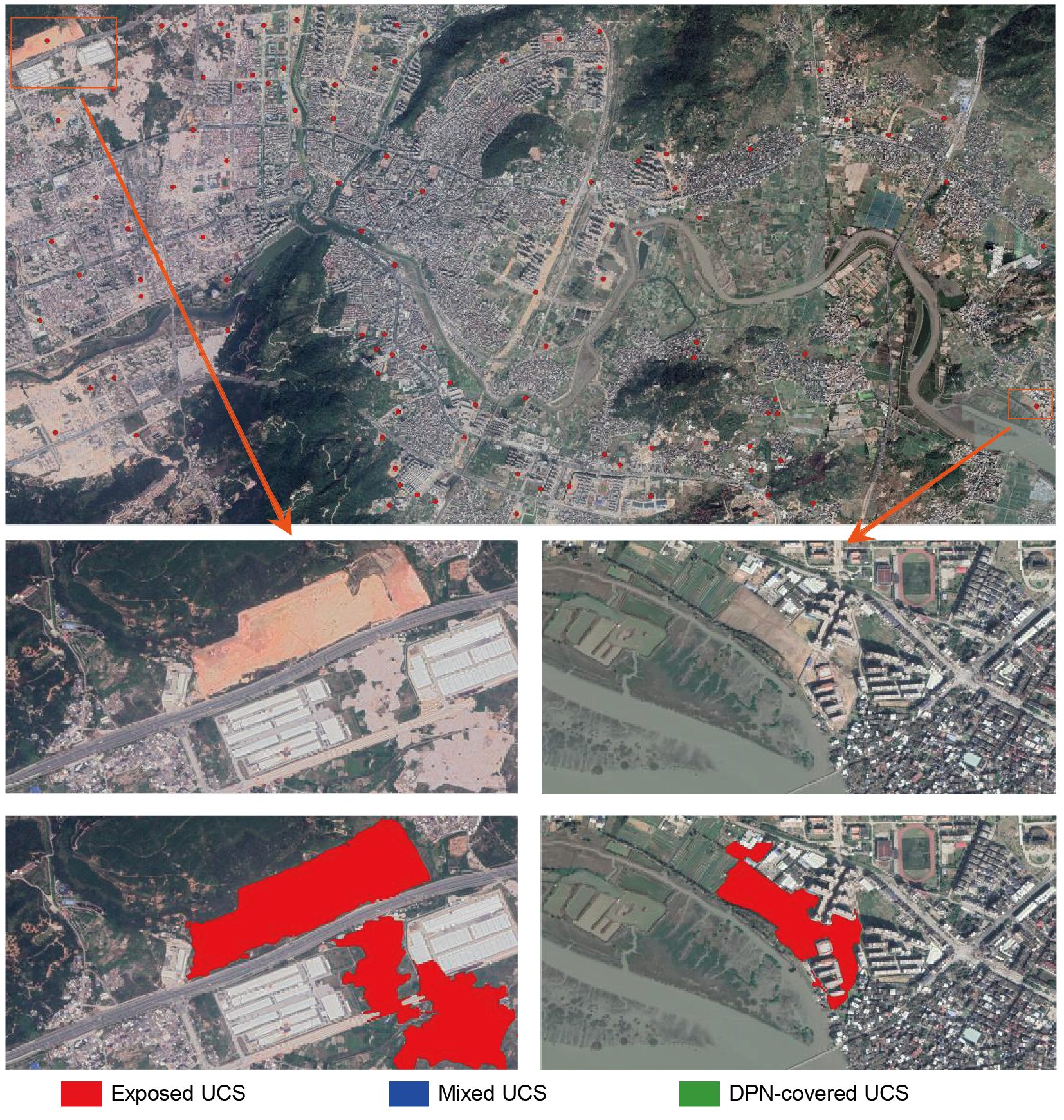


**Supplementary Fig. 22. UCS distribution in the new town of Fuzhou**. Fuzhou is one of the big cities. Image data © Google.


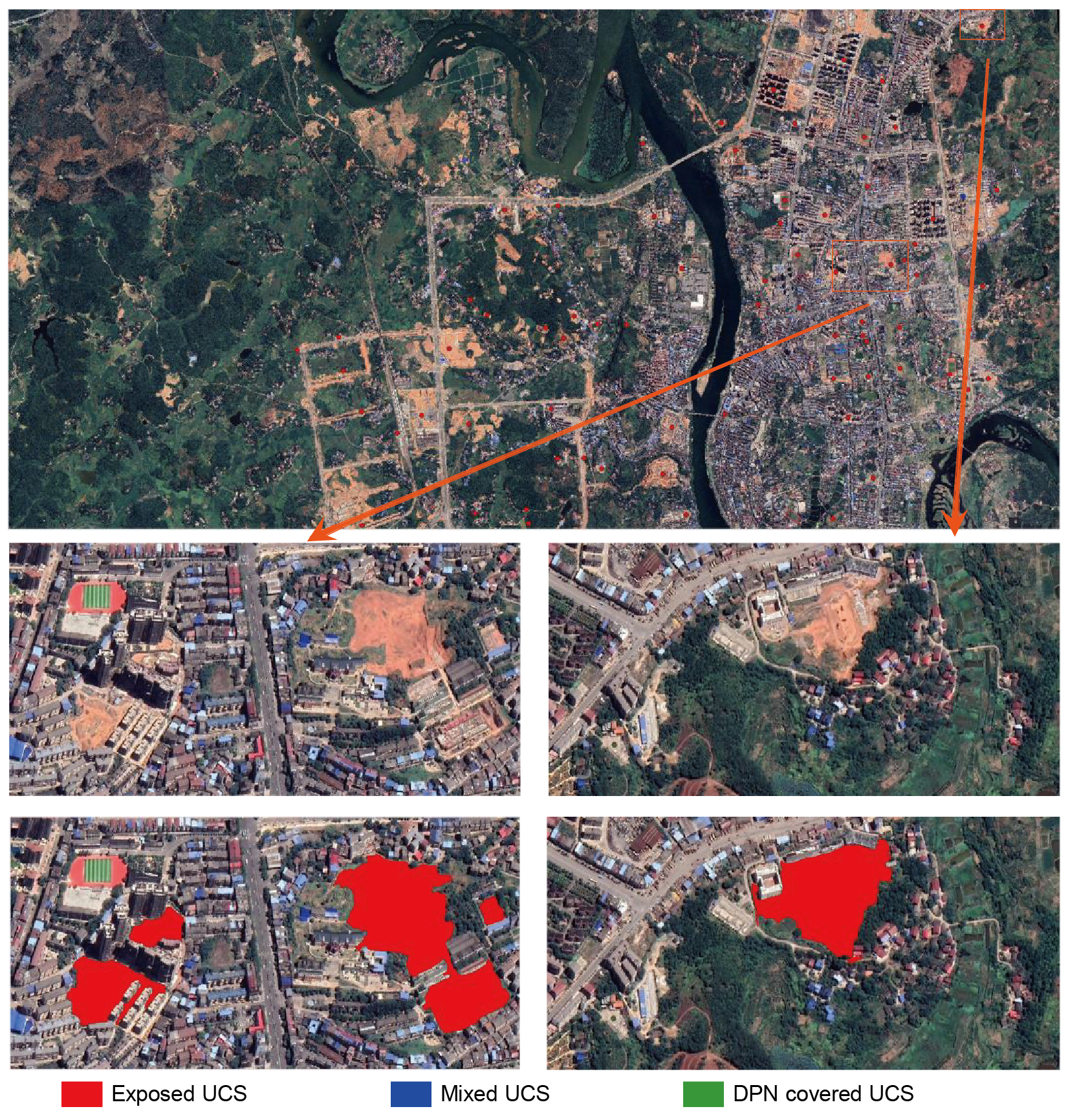


**Supplementary Fig. 23. UCS distribution in the new town of Yongzhou**. Yongzhou is one of the middle cities. Image data © Google.


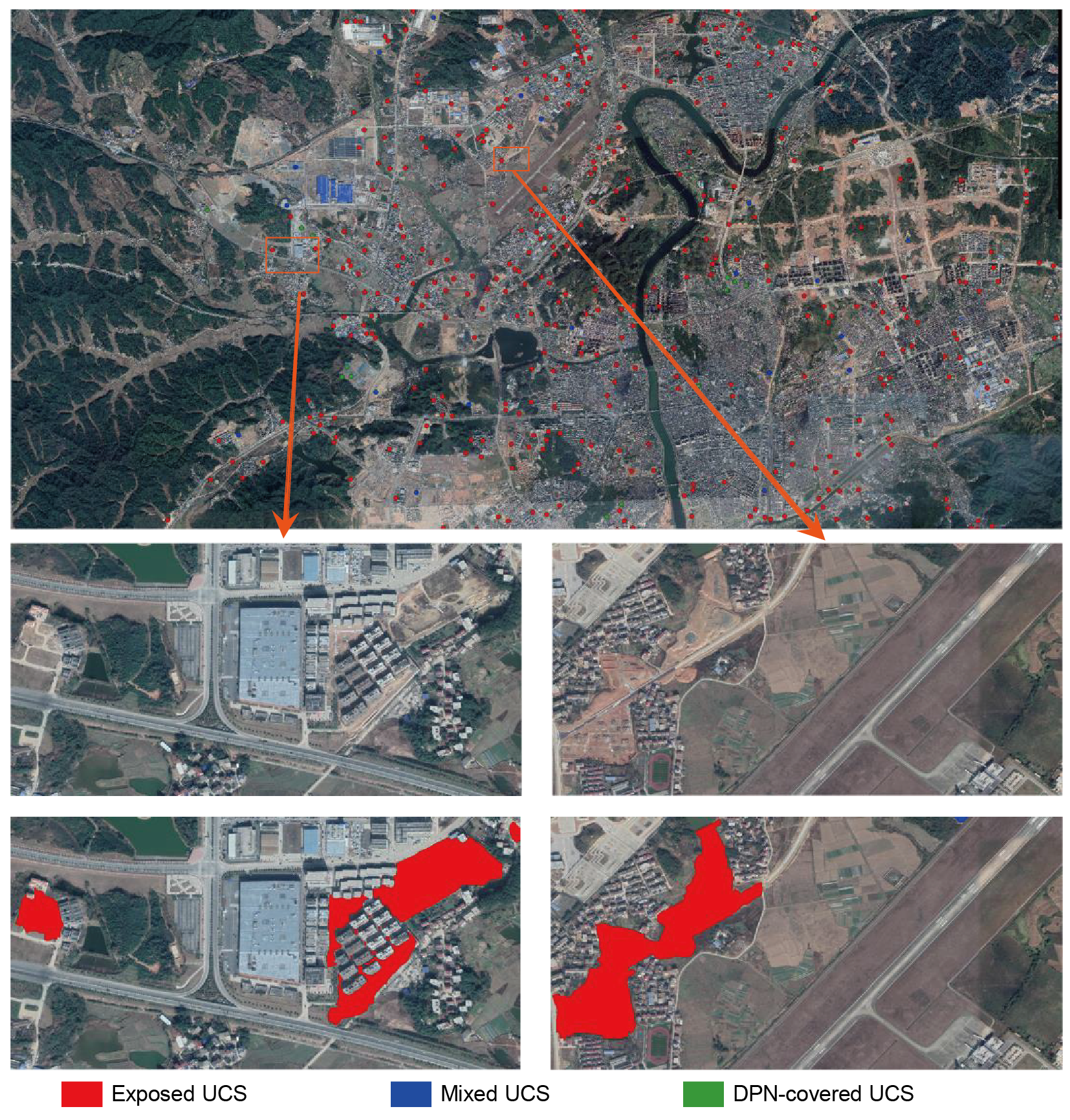


**Supplementary Fig. 24. UCS distribution in the fringe of Jingdezhen.** Jingdezhen is one of the middle cities. Image data © Google.


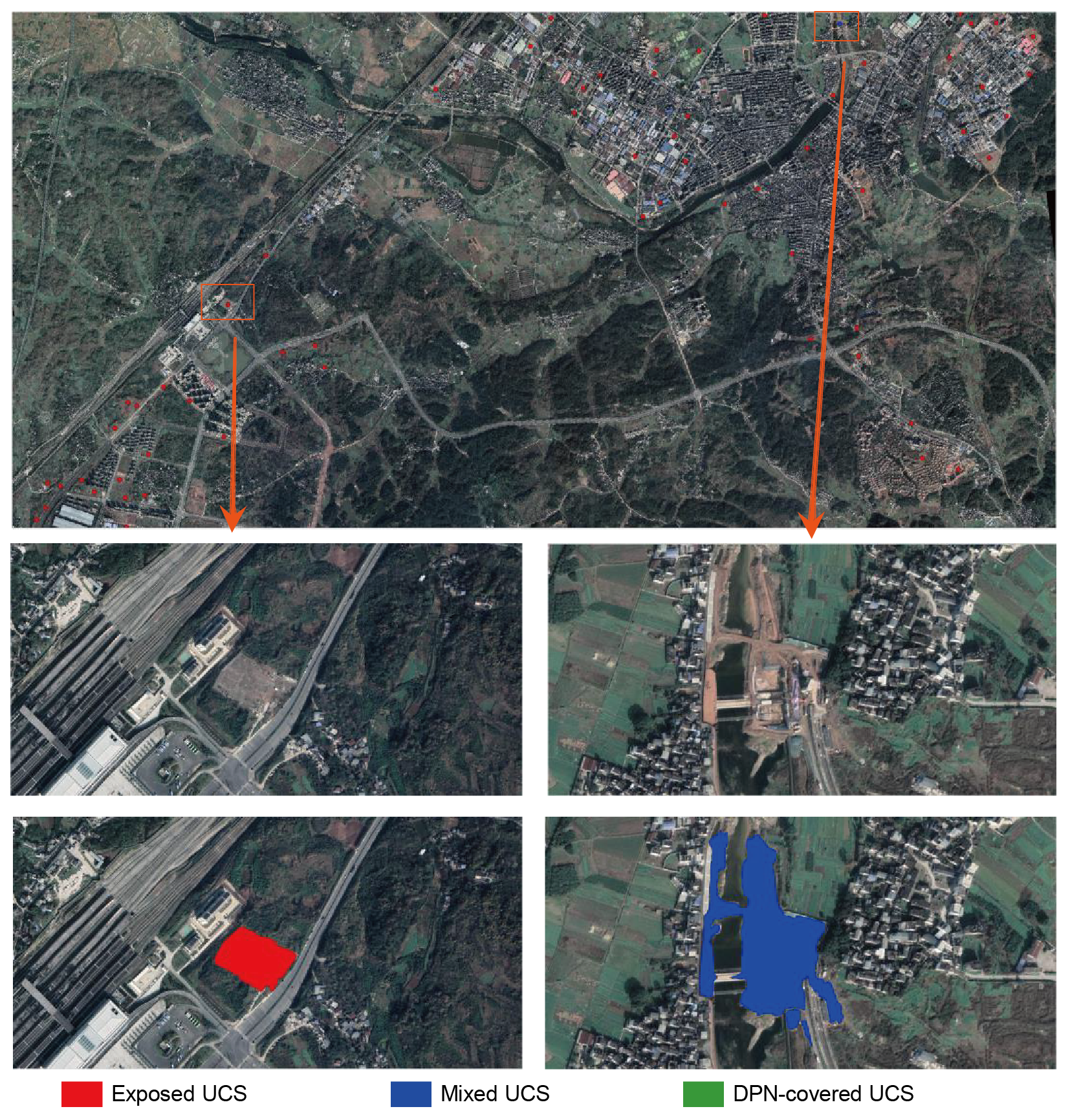


**Supplementary Fig. 25. UCS distribution in the fringe of Huangshan**. Huangshan is one of the small cities. Image data © Google.


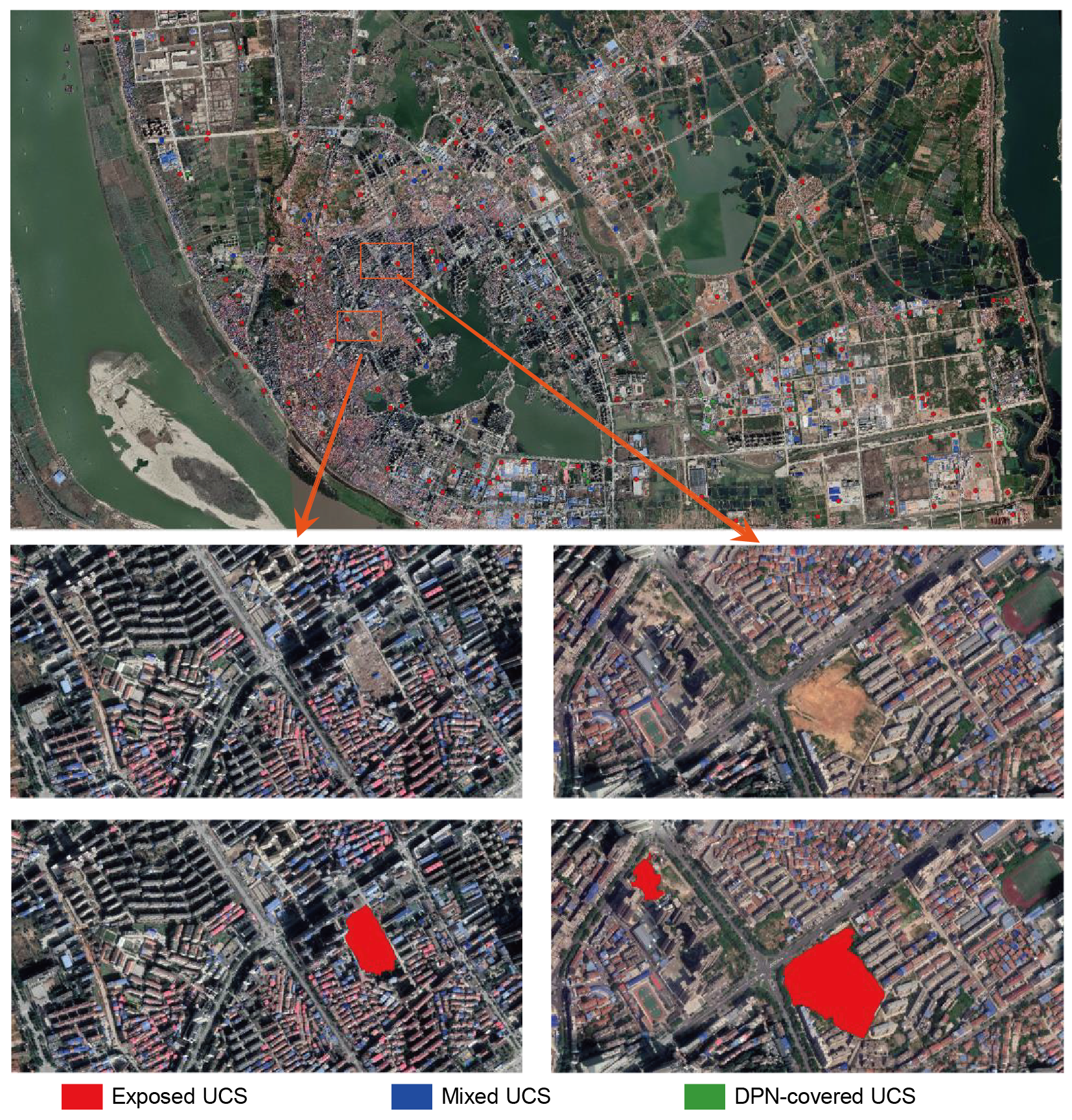


**Supplementary Fig. 26. UCS distribution in the core of Huanggang**. Huanggang is one of the small cities. Image data © Google.

**Supplementary Table 1. Comparison of computational efficiency among different models.**

| **Model** | **Inference speed (FPS)** | **Parameters (Million)** | **FLOPs (GFLOPs)** |
| --- | --- | --- | --- |
| MDCNN | 47.17 | 3.93 | 28.93 |
| HRNet | 17.72 | 28.78 | 54.20 |
| ParC-Net-S | 51.18 | 5.81 | 3.58 |
| TopFormer-B | 94.70 | 5.05 | 2.67 |
| STTBNet | 36.59 | 27.98 | 31.28 |

**Supplementary Table 2. The comparison of accuracy among different models.**

| Model | Exposed Areas (%) | | DNP-covered Areas (%) | | Building (%) | | Background (%) | | OA (%) |
| --- | --- | --- | --- | --- | --- | --- | --- | --- | --- |
|  | **Sens.^*^** | **Prec.^*^** | **Sens.** | **Prec.** | **Sens.** | **Prec.** | **Sens.** | **Prec.** |  |
| MDCNN | 72.1 | 79.8 | 82.9 | 83.3 | 79.1 | 84.4 | 93.3 | 90.3 | 87.9 |
| HRNet | 77.4 | 82.8 | 82.7 | 93.9 | 85.2 | 89.5 | 94.7 | 92.2 | 90.6 |
| ParC-Net-S | 59.9 | 79.4 | 71.0 | 87.6 | 59.3 | 80.8 | 94.5 | 85.0 | 84.0 |
| TopFormer-B | 69.6 | 82.6 | 79.9 | 92.7 | 75.1 | 86.5 | 94.8 | 89.1 | 88.0 |
| STTBNet | 83.5 | 82.6 | 90.2 | 93.4 | 88.2 | 91.9 | 94.8 | 94.2 | 92.1 |

* Sens. and Prec. are short for Sensitivity and Precision respectively.

**Supplementary Table 3. The performance of STTBNet with and without merge module.**

| **Fusion module** | **OA (%)** | **Exposed Areas (%)** | | **DNP-covered Areas (%)** | | **Building (%)** | | **Background (%)** | |
| --- | --- | --- | --- | --- | --- | --- | --- | --- | --- |
|  |  | **Sens.^*^** | **Prec.^*^** | **Sens.** | **Prec.** | **Sens.** | **Prec.** | **Sens.** | **Prec.** |
| **×** | 91.3 | 80.6 | 81.1 | 89.2 | 92.9 | 87.2 | 92.0 | 94.6 | 93.4 |
| Baseline | 91.8 | 80.9 | 83.3 | 92.0 | 92.0 | 87.8 | 92.2 | 95.0 | 93.6 |
| CBAM | 91.6 | 83.5 | 80.3 | 90.2 | 93.3 | 86.9 | 92.7 | 94.3 | 93.9 |
| SE block | 91.9 | 82.6 | 82.8 | 88.6 | 94.0 | 87.5 | 91.9 | 94.9 | 93.8 |
| CrissCross Attention | 91.9 | 81.2 | 83.3 | 88.8 | 94.8 | 88.7 | 91.5 | 94.9 | 93.7 |
| Proposed work | 92.1 | 83.5 | 82.6 | 90.2 | 93.4 | 88.2 | 91.9 | 94.8 | 94.2 |

* Sens. and Prec. are short for Sensitivity and Precision respectively.

**Supplementary Table 4. Sensitivity analysis of loss-weighting schemes**

| **Loss weight**  $\lambda_{rgb}$:$\lambda_{merge}$ | **Exposed Areas (%)** | | **DNP-covered Areas (%)** | | **Building (%)** | | **Background (%)** | |
| --- | --- | --- | --- | --- | --- | --- | --- | --- |
|  | **Sens.^*^** | **Prec.^*^** | **Sens.** | **Prec.** | **Sens.^*^** | **Prec.^*^** | **Sens.** | **Prec.** |
| 1:1 | 83.5 | 82.6 | 90.2 | 93.4 | 88.2 | 91.9 | 94.8 | 94.2 |
| 2:1 | 80.1 | 84.0 | 91.1 | 92.3 | 88.1 | 92.3 | 95.3 | 93.5 |
| 1:2 | 82.4 | 82.6 | 91.2 | 92.0 | 87.3 | 91.7 | 94.7 | 93.8 |
| 3:1 | 82.7 | 82.4 | 90.5 | 93.8 | 88.7 | 91.5 | 94.6 | 94.0 |
| 1:3 | 82.4 | 82.2 | 91.5 | 92.0 | 86.9 | 91.6 | 94.6 | 93.7 |
| 10:1 | 81.3 | 83.1 | 90.0 | 93.5 | 88.1 | 91.9 | 94.9 | 93.7 |
| 1:10 | 80.9 | 82.5 | 89.4 | 93.8 | 87.0 | 90.8 | 94.7 | 93.3 |
| 100:1 | 82.3 | 81.6 | 89.6 | 94.1 | 88.0 | 92.2 | 94.6 | 93.8 |
| 1:100 | 81.1 | 82.7 | 90.1 | 93.2 | 86.0 | 90.3 | 94.6 | 93.3 |

* Sens. and Prec. are short for Sensitivity and Precision respectively.

**Supplementary Table 5. Stratified UCS segmentation performance by city size: class-wise sensitivity/precision and overall accuracy (OA) on the 500-tile national model test set.**

| **City size** | **Exposed Areas (%)** | | **DPN-covered Areas (%)** | | **Building (%)** | | **Background (%)** | | **OA (%)** |
| --- | --- | --- | --- | --- | --- | --- | --- | --- | --- |
|  | **Sens.^*^** | **Prec.^*^** | **Sens.** | **Prec.** | **Sens.** | **Prec.** | **Sens.** | **Prec.** |  |
| Small cities (90 tiles) | 90.0 | 87.9 | 83.1 | 98.8 | 92.3 | 97.3 | 96.6 | 96.2 | 94.8 |
| Medium cities (124 tiles) | 86.8 | 84.5 | 89.2 | 96.5 | 92.0 | 94.8 | 95.4 | 95.4 | 93.5 |
| Large cities (158 tiles) | 82.0 | 82.2 | 93.2 | 96.6 | 89.7 | 94.2 | 95.5 | 94.4 | 92.8 |
| Megacities (68 tiles) | 79.5 | 82.8 | 84.4 | 93.0 | 91.3 | 94.3 | 95.0 | 93.1 | 91.7 |
| Super-cities (58 tiles) | 80.4 | 76.2 | 94.8 | 89.4 | 89.9 | 93.5 | 94.1 | 94.5 | 92.0 |
| Total: 500 (tiles) | 84.5 | 83.7 | 91.3 | 94.6 | 91.0 | 94.7 | 95.4 | 94.8 | 93.1 |

Note: For samples crossing multiple city sizes, the sample is evaluated once within each corresponding city-size test set. * Sens. and Prec. are short for Sensitivity and Precision respectively.

**Supplementary Table 6. Stratified UCS segmentation performance by urban zone: class-wise sensitivity/precision and overall accuracy (OA) on the 500-tile national model test set.**

| **Urban zone** | **Exposed Areas (%)** | | **DPN-covered Areas (%)** | | **Building (%)** | | **Background (%)** | | **OA (%)** |
| --- | --- | --- | --- | --- | --- | --- | --- | --- | --- |
|  | **Sens.^*^** | **Prec.^*^** | **Sens.** | **Prec.** | **Sens.** | **Prec.** | **Sens.** | **Prec.** |  |
| Urban core (90 tiles) | 80.1 | 80.2 | 93.4 | 96.6 | 89.4 | 93.9 | 95.3 | 94.0 | 92.5 |
| Urban new towns (124 tiles) | 83.5 | 82.6 | 92.9 | 96.9 | 90.5 | 94.6 | 96.1 | 95.3 | 93.7 |
| Urban fringe (394 tiles) | 85.6 | 84.1 | 90.4 | 93.6 | 92.0 | 95.2 | 95.4 | 95.1 | 93.2 |
| Total: 500 (tiles) | 84.5 | 83.7 | 91.3 | 94.6 | 91.0 | 94.7 | 95.4 | 94.8 | 93.1 |

Note: For samples crossing multiple urban zones, the sample is evaluated once within each corresponding urban-zone test set. * Sens. and Prec. are short for Sensitivity and Precision respectively.

**Supplementary Table 7. Cross-sensor validation results across diverse cities and VHR datasets.**

| **City** | **Sensor** | **GSD**  **(m)** | **OA**  **(%)** | **Exposed Areas**  **(%)** | | **DNP-covered Areas (%)** | | **Building**  **(%)** | | **Background**  **(%)** | | |  |
| --- | --- | --- | --- | --- | --- | --- | --- | --- | --- | --- | --- | --- | --- |
|  |  |  |  | **Sens.^*^** | **Prec.^*^** | **Sens.** | **Prec.** | **Sens.** | **Prec.** | | **Sens.** | **Prec.** | |
| Zhuhai | Pleiades | 0.5 | 92.7 | 90.3 | 89.7 | 70.3 | 86.2 | 78.6 | 78.0 | | 95.2 | 95.4 | |
| Shenyang | GF-7 | 0.65 | 93.2 | 88.7 | 89.2 | 81.1 | 92.2 | 90.8 | 90.0 | | 95.3 | 95.1 | |
| Beijing | GF-2 | 1.0 | 89.0 | 78.1 | 81.2 | 70.6 | 76.5 | 79.9 | 85.2 | | 93.7 | 91.9 | |
| Shenzhen | GF-1 | 2.0 | 93.3 | 81.7 | 82.3 | 53.9 | 73.2 | 75.2 | 83.4 | | 96.8 | 95.4 | |
| Xian | ZY-3 | 2.1 | 91.2 | 62.2 | 65.8 | 69.7 | 77.6 | 88.1 | 88.0 | | 95.2 | 94.4 | |

* Sens. and Prec. are short for Sensitivity and Precision respectively.

**Supplementary Table 8. Season-stratified Kruskal–Wallis omnibus tests for target UCS classes.**

| **Target class** | **Metric** | **Spring (*n*)** | **Summer (*n*)** | **Autumn (*n*)** | **Winter (*n*)** | ***H*** | **d*f*** | ***p*-value** | **Decision (*α*=0.05)** |
| --- | --- | --- | --- | --- | --- | --- | --- | --- | --- |
| Exposed areas | Precision | 126 | 66 | 134 | 146 | 5.877 | 3 | 0.118 | Fail to reject H₀ |
|  | Sensitivity | 130 | 64 | 131 | 149 | 3.831 | 3 | 0. 3 | Fail to reject H₀ |
| DPN-covered areas | Precision | 59 | 34 | 61 | 84 | 2.064 | 3 | 0.559 | Fail to reject H₀ |
|  | Sensitivity | 59 | 34 | 63 | 85 | 2.763 | 3 | 0.430 | Fail to reject H₀ |

*Notes.* Seasons are defined by month (Spring: Mar–May; Summer: Jun–Aug; Autumn: Sep–Nov; Winter: Dec–Feb). For each metric, $n$denotes the number of tiles in which the target class is present; *H*_0_: no difference among seasons; *α* = 0.05.

**Supplementary Table 9. Sensitivity of UCS parcel counts by size class in Beijing under different post-processing buffer radii.**

|  | **Number of UCS** | | | | | |
| --- | --- | --- | --- | --- | --- | --- |
|  | **Micro** | **Small** | **Medium** | **Large** | **Mega** | **Total** |
| 10m | 8796 | 4293 | 4945 | 2925 | 358 | 21317 |
| 25m | 4516 | 2623 | 3669 | 2414 | 395 | 13617 |
| 50m | 2190 | 1587 | 2658 | 1871 | 393 | 8699 |
| 100m | 764 | 771 | 1538 | 1293 | 354 | 4720 |
| 200m | 164 | 207 | 497 | 536 | 233 | 1637 |

**Supplementary Table 10. Pixel-level accuracy under different post-processing buffer radii (500-tile nationwide test set).**

| **Buffer** | **OA (%)** | **Exposed Areas (%)** | | **DNP-covered Areas (%)** | | | **Building (%)** | | **Background (%)** | |
| --- | --- | --- | --- | --- | --- | --- | --- | --- | --- | --- |
|  |  | **Sens.^*^** | **Prec.^*^** | **Sens.** | **Prec.** | **Sens.** | | **Prec.** | **Sens.** | **Prec.** |
| 10m | 91.2 | 76.7 | 71.5 | 45.9 | 58.6 | 63.7 | | 90.4 | 97.5 | 93.6 |
| 25m | 94.3 | 88.1 | 82.7 | 86.6 | 91.8 | 80.9 | | 89.9 | 97.1 | 96.2 |
| 50m | 91.5 | 66.7 | 83.4 | 26.0 | 91.1 | 73.8 | | 80.7 | 97.5 | 93.6 |
| 100m | 90.3 | 53.3 | 83.7 | 11.5 | 95.1 | 74.1 | | 71.9 | 97.5 | 93.6 |
| 200m | 88.8 | 33.4 | 84.0 | 6.3 | 99.1 | 74.8 | | 62.1 | 97.4 | 93.8 |

**Supplementary Table 11 Year-to-year UCS mapping in Shenzhen (2022–2023).**

|  | UCS area (km2) | DPN area (km2) | Number of UCS | | | | | |
| --- | --- | --- | --- | --- | --- | --- | --- | --- |
|  |  |  | Micro | Small | Medium | Large | Mega | Total |
| 2022 | 46.55 | 3.36 | 212 | 198 | 513 | 747 | 85 | 1755 |
| 2023 | 41.48 | 2.91 | 274 | 258 | 724 | 645 | 76 | 1977 |

**Supplementary Table 12. Covariate balance diagnostics for national 1-km grid matching**

| **Covariate** | **UCS** | | **DPN** | |
| --- | --- | --- | --- | --- |
|  | **Adjusted SMD** | **Adjusted Variance Ratio** | **Adjusted SMD** | **Adjusted Variance Ratio** |
| Propensity score (distance) | 0.0176 | 1.0509 | 0.0668 | 1.0562 |
| wind speed | 0.0077 | 0.9354 | 0.0350 | 1.2907 |
| temperature | -0.0110 | 0.9975 | 0.0900 | 0.7916 |
| humidity | -0.0003 | 0.8760 | 0.0811 | 0.6986 |
| impervious surface density | -0.0497 | 0.8343 | -0.0413 | 1.2100 |
| DEM | -0.0061 | 0.9178 | -0.0584 | 0.7969 |
| population density | -0.0562 | 0.7639 | -0.0062 | 1.0752 |
| industrial building density | 0.0978 | 1.0624 | -0.0915 | 0.7361 |
| construction site density 2020 | - | - | 0.0288 | 0.5817 |
| traffic density PC1 | 0.0072 | 1.0528 | 0.0228 | 0.4270 |
| traffic density PC2 | 0.0478 | 2.6429 | -0.0082 | 0.3883 |
| road networks PC1 | -0.0260 | 0.8056 | 0.0143 | 0.8293 |
| road networks PC2 | -0.0593 | 0.7433 | 0.0094 | 1.0508 |
| road networks PC3 | -0.0292 | 0.8985 | -0.0393 | 0.6190 |

Notes: Adjusted SMD stands for Adjusted Standardized Mean Difference. The caliper was set to 0.15 for the UCS matching and 0.10 for the DPN matching.

**Supplementary Table 13 Descriptions of city-level UCS indicators**

| **Abbreviation** | **Description** |
| --- | --- |
| Number of Medium UCS | Number of medium UCSs with area 1,600 m²–1 ha (10,000 m²) (unit: sites). |
| Number of Large UCS | Number of large UCSs with area 1–10 ha (unit: sites). |
| Number of Mega UCS | Number of very large UCSs with area > 10 ha (unit: sites). |
| Population | Total city population within the urban extent defined by GUB and its refined version GUSV, aggregated from a 100 m gridded population dataset (unit: persons; visualized on a log₂ scale, 2^x^, in Fig. 3h). |
| Density of Micro UCS | Density of very small UCSs (< 400 m²), defined as the Number of Micro UCS / the area of city(unit: sites/km²). |
| Density of Small UCS | Density of small UCSs (400–1,600 m²), defined as the Number of Small UCS / the area of city (unit: sites/km²). |
| The area of city | Urban area of the city in 2020, calculated from the Global Urban Boundary (GUB) and its refined version GUSV (unit: km²). |
| Proportion of Mega sites in UCSs > 1600 m^2^ | Proportion of mega sites (> 10 ha) among all UCS sites larger than 1,600 m² (unit: %, implemented as a 0–1 fraction in the analysis). |

**Supplementary Table 14: Kruskal–Wallis Test for Housing Construction Area and Housing Construction Density Across City Typologies.**

| **Item** | **Value** |
| --- | --- |
| Outcome variable | Housing construction area (10 000 sq.m), Housing construction density (%) |
| Groups (*k*) | 4 typologies: Integrated renewal (n=58), Stock-renewal transition (n=196), Micro-renewal focus (n=73), Low-intensity maintenance (n=18) |
| Total sample size (*N*) | 345 cities |
| Test statistic (*H*) | 61.941 |
| Degrees of freedom (d*f*) | 3 |
| *p*-value (two-sided) | < 0.001 |
| Decision (*α* = 0.05) | Reject *H*₀ (distributions differ across groups) |
| *Notes:* Housing construction area statistics were compiled from municipal statistical yearbooks[S43] [S44]. Follow-up pairwise comparisons use Holm-adjusted *p*-values (see Supplementary Table 15). | |

**Supplementary Table 15: Pairwise Mann–Whitney tests with Holm adjustment for housing construction area and construction density across the four city typologies.**

| **Pairwise comparison** | **Mann–Whitney U** | **Adjusted p-value** | **Decision (α = 0.05)** |
| --- | --- | --- | --- |
| Integrated renewal vs Low-intensity maintenance | 62.0 | < 0.001 | Significant |
| Integrated renewal vs Micro-renewal focus | 766.0 | < 0.001 | Significant |
| Integrated renewal vs Stock-renewal transition | 3405.0 | < 0.001 | Significant |
| Low-intensity maintenance vs Micro-renewal focus | 963.0 | 0.0023 | Significant |
| Low-intensity maintenance vs Stock-renewal transition | 2926.0 | < 0.001 | Significant |
| Micro-renewal focus vs Stock-renewal transition | 9046.0 | 0.0017 | significant |
| Notes: *p*-values are already Holm-adjusted for six pairwise tests. Directional interpretation is consistent with the descriptive group statistics reported elsewhere in the Supplementary. | | | |

**Supplementary Table 16. Cluster-wise partial correlations (small-UCS density vs. number of medium UCSs) with standard errors and 95% confidence intervals (corresponding to Fig. 3b; controls: urban area).**

| **Cluster name (No.)** | **Number** | **Partial *r*** | **Partial *p*** | **Partial SE** | **Partial CI_low_** | **Partial CI_high_** |
| --- | --- | --- | --- | --- | --- | --- |
| Integrated renewal cities (1) | 59 | 0.907 | <0.001 | 0.134 | 0.847 | 0.944 |
| Low-intensity maintenance cities (2) | 29 | 0.702 | <0.001 | 0.196 | 0.452 | 0.850 |
| Micro-renewal focus cities (3) | 78 | 0.448 | <0.001 | 0.115 | 0.251 | 0.610 |
| Stock renewal transition cities (4) | 205 | 0.527 | <0.001 | 0.070 | 0.420 | 0.619 |
